# Novel multimodal mechanical stimulation is superior to TENS to treat and prevent chronic low back pain: a randomized controlled trial

**DOI:** 10.3389/fpain.2025.1625420

**Published:** 2025-08-18

**Authors:** Amy Lynn Baxter, Jena L. Etnoyer-Slaski, Owen Tucker, Jessica Allia Rice Williams, Kevin Swartout, Lindsey L. Cohen, M. Louise Lawson

**Affiliations:** ^1^ Department of Emergency Medicine, Augusta University, Augusta, GA, United States; ^2^ Harmonic Scientific LLC (parent company MMJ Labs LLC), Lewes, DE, United States; ^3^ Kaizo Clinical Research Institute, Landover, MD, United States; ^4^ Emory University, Atlanta, GA, United States; ^5^ Department of Health Policy and Administration, Penn State College, University Park, PA, United States; ^6^ Department of Psychology, Georgia State University, Atlanta, GA, United States; ^7^ Department of Statistics and Analytical Sciences, Kennesaw State University, Kennesaw, GA, United States

**Keywords:** mechanical low back pain, prevention, focal mechanical vibration, PROMIS (patient-reported outcomes measurement information system), spine biomechanics, vibration, acute low back pain (aLBP), chronic low back pain (cLBP)

## Abstract

**Background:**

Low back pain (LBP) is the leading cause of disability worldwide. Up to half of moderate-to-severe acute LBP (aLBP) progress to chronic (cLBP), with neuromotor, fascial, and muscle pathology contributing to inoperable mechanical disability. A novel thermomechanical stimulation (M-Stim) device delivering stochastic and targeted vibration frequencies relieved LBP in a pilot. Efficacy versus an active control, for cLBP prevention, or reversing disability was undetermined.

**Methods:**

As part of a National Institutes of Health (NIH) double-blind, randomized controlled trial, 159 chiropractic patients with non-radiating moderate-to-severe LBP [Numeric Rating Scale (NRS) ≥4] were randomized to add either the multimodal M-Stim device or 4-lead transcutaneous electrical nerve stimulation (TENS) for 30 minutes daily to other therapies. Between June 2022 and July 2024, pain scores, analgesic use, and device adherence were recorded for 28 days, with weekly follow-up up to 6 months. Primary outcomes included PROMIS Pain Interference scores, NRS pain scores, and transition from aLBP to cLBP (Pain Interference ≥55 at 3 months). Exploratory analyses examined higher-severity subgroups, including those meeting NIH Research Task Force (RTF) criteria, obesity, longer pain duration, and an integrated analysis with common criteria for intractable inoperable mechanical cLBP.

**Results:**

For 44 aLBP and 115 cLBP participants [mean age 42.6, 54% female, BMI 30.9 (SD 6.19), NRS 5.51 (SD 2.15)], M-Stim was noninferior to TENS for initial and 10-day relief. Over time, Linear Mixed Models (intention-to-treat) showed M-Stim significantly improved pain and disability for both aLBP and cLBP, (*p* < .001 to *p* = .024). With higher severity, 23.9% (11/46) M-Stim users reached “no disability” (PROMIS = 40.7) vs. 7.1% (2/28) TENS users [RR 0.81 (95% CI 0.66–0.99), *p* = 0.04]. M-Stim yielded significantly greater improvement than TENS in those with pain ≥5 years, BMI ≥30, or mechanical cLBP (all *p* < .05). Significantly fewer aLBP M-Stim users transitioned to cLBP at 3 months [31.8% vs. 72.7%, RR 0.44 (95% CI 0.23–0.85), NNT = 2.4, *p* = 0.015].

**Conclusions:**

A multimodal M-Stim device reduced progression to cLBP significantly more than TENS. Both devices reduced pain initially, but M-Stim reduced pain and disability significantly more over time, particularly in cLBP subsets with higher severity, duration, or BMI.

**Clinical Trial Registration:**

https://clinicaltrials.gov/study/NCT04494698, identifier NCT04494698.

## Introduction

Low back pain (LBP) is the most disabling condition worldwide, responsible for over 70 million years lived with disability annually (1). Up to 80% of adults will experience acute LPB (aLBP) in their lifetimes, with up to 50% of moderate-to-severe aLBP becoming chronic (cLBP ≥3  m) (2). Opioid use, body mass index (BMI), female sex, and psychological factors are associated with chronicity. Increasingly, paraspinal muscles, postural instability and thoracolumbar fascial derangement are viewed as targets for intervention (3–5). Within days of severe muscular, ligamentous, bony, or nerve injury, compensatory multifidus and erector spinae muscular derangement begins. The injured muscles undergo a pattern of inflammation, reactive hypertrophy, hypoperfusion, and spasm (3, 6). External or pain-mediated immobilization leads to muscular fatty changes and further hypoperfusion within weeks (7, 8), with inflammatory changes in fascia causing pain and instability as pain transitions to chronic (5, 9, 10). Reflex and proprioceptive responses to pain are altered, with this neuromotor hypofunction (11) associated with ongoing functional instability and pain (12, 13). While previously these nonspecific findings in “mechanical” cLBP meant non-treatable (14), interventions preserving or improving muscle and fascia function (15) could potentially reduce acute-to-chronic transition as well as reverse the disability of mechanical cLBP.

Evidence-based guidelines recommend multimodal pain interventions for both acute exacerbations and chronic rehabilitation (16–18). Physical modalities including cold reduce inflammation. Heat reduces spasm and increases perfusion, releasing adhered fascia that painfully restricts movement (19). Exercise and yoga reduce acute inflammatory pain and improve cLBP (20), transcutaneous electrical nerve stimulation (TENS) is well-established to reduce pain intensity for aLBP via central endogenous opioid release (21, 22), and acupuncture and acupressure are well-supported (23).

Two emerging “precision physics” therapies (vibration and implanted electrical stimuli) may restore function for mechanical cLBP. In addition to blocking transmission of pain via the 200 Hz neuromodulatory frequency (24), focal mechanical stimulation (M-Stim) at other frequencies reduces LBP via various hypothesized mechanisms (25–27). Whether through pain inhibition, improving proprioception (28), or restoring neuromotor function (29), the initial common pathway likely involves newly described mechanical force ion channels repairing myofascial contributors to mechanical cLBP (25, 27, 30). Recently, twice daily electrical stimulation (E-Stim) via electrodes implanted in the multifidus muscle reduced mechanical cLBP disability from severe to moderate in 6 months (31), down to mild within 2 years (32). The authors attribute the improvement in part to improved neuromotor control (33), which is also a fast-acting effect of vibration well-described in kinesiotherapy literature (29).

An NIH-funded multimodal heat, pressure, and harmonic multifrequency vibration device (M-Stim) reduced both a/cLBP 57% after 20 minutes in a recent pilot (34). Neither the multifidus E-Stim nor the M-Stim have been tested over time against a control. This prospective, randomized active-controlled trial investigated pain, disability, and chronicity progression in moderate-to-severe LBP subjects using M-Stim or TENS. The objectives were to compare immediate and 10-day Pain Intensity using a numeric rating scale (NRS), and cLBP disability using PROMIS Pain Interference. Outcomes included 3-month resolution of aLBP and 6-month cLBP restoration of normal function (Pain Interference < 55), and exploratory outcomes in more at-risk or severely affected subjects. A neural network identified characteristics associated with M-Stim responders.

## Methods

### Device description

**Figure 1:**
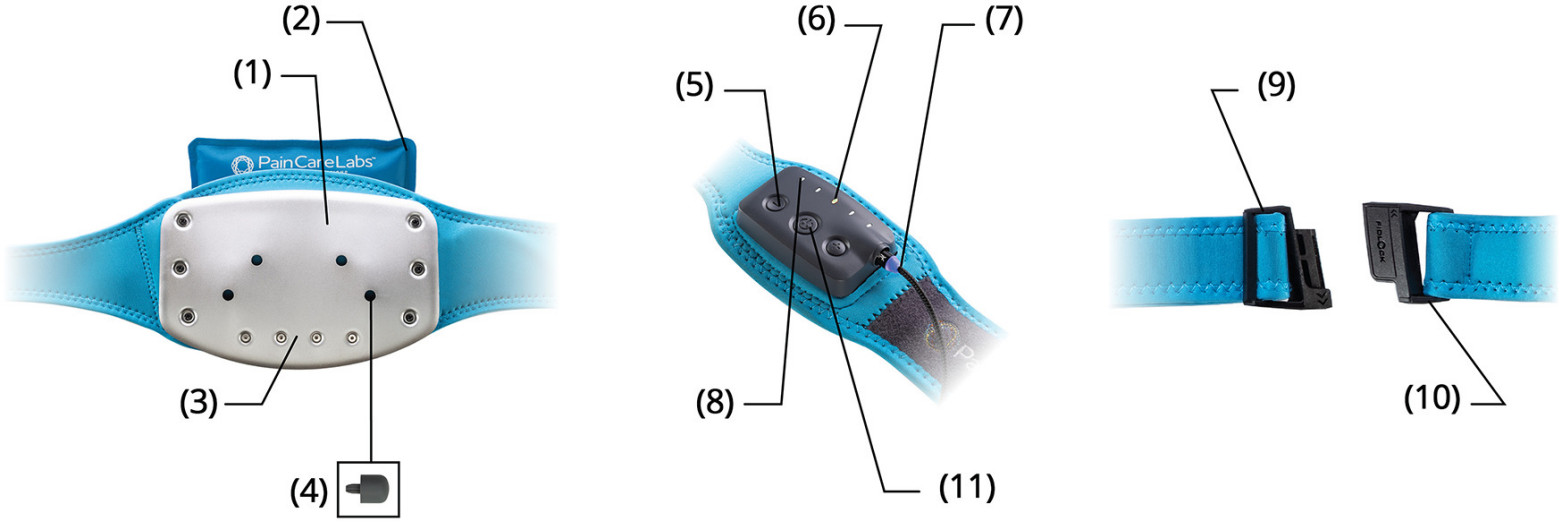
Contoured temperature plate (1). Natural Clay Ice/Heat Pack (2). Multi-Vibration Motor Array (3). Trigger Point Acupressure Nubs (4). Five Intensity Settings (5). LED Cycle & Intensity Display (6). Magnetic Charging Cable (7). Eight Therapy Cycles (8). Custom Fit Waistband (9). Slide-N-Lock Magnetic Buckle (10). Haptic Touch Panel (11).

Mechanical low back pain dysfunction typically affects multiple vertebral lengths of muscle and a larger area of overlying fascia (10, 19). To cover the thoracolumbar field, the multimodal M-Stim device (DuoTherm™, Harmonic Scientific LLC, Lewes, DE) is a wearable 13 × 20 cm thermoconductive metal plate held by a compressive neoprene belt stretching to 150 cm. Harmonic motor frequencies (50 Hz, 100 Hz, and 200 Hz) deliver stochastically varying patterns and beats of mechanical (vibratory) force (34). To direct the impulses, the DuoTherm device incorporates metal shaping to concentrate pressure on the paraspinal muscles while applying varying amplitudes of M-Stim to the lumbar fascia field (Figure 1). The approach was first described by Lundeberg, who found a single 100 Hz or 200 Hz motor on a 6″ × 8″ flat plate reduced low back pain more than TENS, with some vibration subjects experiencing a prolonged pain reduction of days to weeks (35, 36).

Eight patterns or “therapy cycles” couple temporary nociceptor neuromodulation pain blockade with frequencies associated with different cellular repair processes. Over 100 studies of a single-motor 200 Hz device demonstrate significant a-delta nociceptor pain reduction (37, 38). The mechanism, described by Salter et al, is driven by Pacinian mechanoreceptors maximally inhibiting nociceptor firing through dorsal horn ATP release and adenosine blocking of the presynaptic spinothalamic tract (“spinal gating”) (24). Additional pain relief therapy cycle frequencies are associated with reduced calcitonin gene-related peptide (CGRP) in the dorsal root ganglia, oxytocin release inhibiting pain via periaqueductal gray pathways, and reduced delayed onset muscle soreness peripherally and centrally (27). Frequencies associated with mechanical tissue restoration [neuromotor reflex (29, 36), reduction of fatty changes (39), inflammation (40), and vasodilation (41, 42)] are coupled to reach deeper tissues with constructive interference. The 12 therapy cycles programmed for the pilot were reduced to the 8 most frequently used, relying on subject biofeedback to choose patterns (30, 40).

To enhance patient choice and add synergistic tissue and central pain benefits, heat and cold packs can be placed behind the plate to reduce spasm or inflammation. Four holes in the plate allow for different locations of a 1.5 cm silicone acupressure nub to target myofascial trigger points (43), with 5 patient-controlled amplitude settings. Having options improves self-efficacy (feeling empowered to control problems like pain) which is associated with opioid reduction and improved chronic pain management (44).

### Trial design and participants

This prospective randomized double-blind active-controlled trial recruited 160 adults between 20 and 75 years verbally endorsing moderate-to-severe LBP [Numeric Rating Scale (NRS) ≥4/10] between June 2022 and December 2023, with follow-up completed July 2024. Two chiropractic clinics in Maryland and Virginia served as sites to eliminate the potential bias of onsite opioid prescribing. Enrollment was stratified by participant endorsement of chronic (cLBP) ≥3 months (*n* = 100) or acute (aLBP) <3-month pain (*n* = 60) and consenting to 6- and 3-month follow-up, respectively. Exclusion criteria included radicular pain, sickle cell disease, sensitivity to cold or vibration, a pacemaker, diabetic neuropathy or skin lesions in the low back, or inability to apply the devices as directed (Supplementary Material S1).

This trial was part of the National Institutes of Health Help End Addiction Long term (HEAL) program and was funded by the National Institute on Drug Abuse. The Kaizo Clinical Research Institutional Review Board approved the trial, which was registered with ClinicalTrials.gov NCT04494698.

### Interventions

Participants were randomized to add 30-minute daily use of a prescription 8-channel 4-lead electrical stimulation TENS unit (LG Smart TENS, LGMedSupply, Cherry Hill, NJ), or multimodal 8-cycle M-Stim (DuoTherm™, Harmonic Scientific LLC, Lewes, DE) to any ongoing therapies or treatments. Pain, opioid and device use were reported daily for 28 days, and weekly for up to 6 months. (Opioid outcomes beyond first 28-day use are reported elsewhere.).

### Randomization, procedure, blinding, and assessment

Prior to treatment, clinic intake staff assessed eligibility and obtained digital informed consent for a study “to evaluate the effect of an electric or mechanical stimulation device on opioid use and pain relief”. After signing the tablet, the study ID and coded device assignment were randomly generated through a link to a Qualtrics random number generator with no blocking or further stratification. While the participant recorded current pain intensity, study staff retrieved the assigned device. Participants watched the appropriate training video (Supplementary Material S3) and used the device for 30 min while completing registration data entry.

Outcome assessments were completed by the participants, who were blinded to which device powered the study hypotheses. The protocol statistician (KS) and study coordinator (JS) knew device assignments and had access to data, but did not conduct analysis. The PI(AB) was blinded to allocation and all data during enrollment, accessing data only after study completion. The analyzing statisticians (JW, OT) were blinded to device assignment until completion of primary analysis. Success of participant blinding was tested at 3 months with prompts, “select if you think you received… control or treatment” and “How confident are you?”

### Measures

Registration data included the NIH Minimum Data set for low back pain studies (45), (Supplementary Material S3), including demographic information, work and lawsuit status, opioid use for back pain, Sullivan Pain Catastrophizing scale [0(none)-52(extreme)] (46), and Patient-Reported Outcomes Measurement Information System (PROMIS®) (47)Physical Function (4a), Depression (4a), and Pain Interference (8a) (disability) scales. PROMIS responses from 1(low)-5(high) are normed to a United States average T-score where M = 50 SD = 10. For Pain Interference the summed responses from 8 questions yield possible T-scores from no disability (40.7) to completely disabled (77), where mild disability ≥55. (www.healthmeasures.net) We also collected back pain etiology and a 13-intervention inventory of prior treatments including cannabis and gabapentin. (Supplementary Material S3) Clinic staff documented treatments received the day of enrollment, with subjects reporting thereafter.

Text and email prompts reminded participants to record pain (NRS), opioid use, treatments, and device use daily for 28 days, with Pain Interference weekly throughout follow-up. To collect heterogeneous opioid information, we created a skip-logic data collection instrument algorithm [34 dose-per-pill options, 15 opioid formulations (e.g., hydrocodone, hydromorphone), and 6 pill sources], translated to milligrams of morphine equivalents (MME) (Supplementary Material S2, DOSE Tool).

### Outcomes

Outcomes of interest included 30 min and 10-day changes in Pain Intensity using a 0–10 NRS with a clinically significant difference of 2. Pain Interference (disability) were compared by 10 (one SD) and 20 (2 SD) point improvements, resolution to below mild disability T ≥ 55, and elimination of disability (T = 40.7) (48). For spine and LBP, a minimum clinically important difference (MCID) is 8–9 (49). To compare PROMIS Pain Interference to the Oswestry Disability Index (ODI) specifically for LBP, T-scores of 57.7–65.4 correspond to “moderate” LBP disability (ODI of 31–40), and 65.7–71.5 crosswalks to an ODI of “severe” (41–60) (50). Resolution of cLBP disability and avoiding aLBP progression to cLBP were defined by lack of ongoing mild Pain Interference (T ≥ 55) at 6 and 3 months respectively (48).

Exploratory cLBP disability outcomes included subsets with BMI > 30, ongoing pain duration ≥5 years, and two definitions of greater severity. In 2015, Deyo et al. with the NIH Research Task Force (RTF) on Low Back Pain (45) found 7-day average Pain Intensity, PROMIS Physical Function(4a), Depression, and four Pain Interference(4a) questions as most predictive of greater disability, with scores >27 deemed moderate and 36–48 severe in a population being evaluated for spinal surgery. For “intractable inoperable mechanical cLBP”, the FDA defined intractable as attempting 3 or more interventions without pain resolution, and the common severity criteria with Gilligan and Decker's implanted multifidus stimulator studies (31, 32, 51), including pain >6 months, pain more than half the days each week, LBP worse than other pain, and a Pain Interference T-score ≥60 [crosswalked to an Oswestry Disability Index (ODI) of 25] (50), with exclusion criteria of ongoing lawsuit or “psychiatric unacceptability” (PROMIS Depression T-Score ≥60, Pain Catastrophizing ≥50).

Finally, to determine factors associated with improved function after M-Stim, we developed an ensemble machine learning approach combining multiple neural networks. We integrated characteristics from the Low Back Pain Minimum Data Set along with factors associated with LBP chronicity. To predict outcomes, we implemented an ensemble strategy averaging predictions from twenty individual neural networks. The model incorporated early stopping to prevent overfitting. SHAP (SHapley Additive exPlanations) analysis identified key predictive features (Supplementary Material S6).

### Sample size calculation

Sample sizes were initially calculated for the opioid study against standard care: 60 aLBP/opioid naive subjects for initiation outcomes and 100 cLBP with or without chronic opioid use for change over time, based on an effect size of.6 for an implanted spinal cord stimulator on opioid use reduction (52). To estimate power of this intended recruitment, for cLBP pain intensity focal vibration has a SMD of −1.07, while vibration studies for disability show contradictory effect sizes (25). Using the focal vibration effect size estimate of 1.0, a two-tailed test of pain intensity for acute patients with 27 in each group with attrition of 10% (60 enrolled) would give a power of 0.95, or establish noninferiority against TENS (effect size 0.69) (53). Using an effect size of 0.5 for change in disability, 64 participants would be needed for each group with power of 0.8 and significance set at 0.05, indicating the acute group was anticipated to be underpowered for disability. G*Power (54).

### Analysis

Intention-to-treat analysis included summary statistics (means, standard deviations, proportions) calculated using T-tests and relative risks using Chi-squared tests or Fisher's Exact for small cell numbers. For overall pain changes over time, a linear mixed-effects model (LMM) with full-information maximum likelihood followed intention-to-treat principles, with missing data assumed to be at random. NRS Pain Intensity and PROMIS Pain Interference differences at 3- and 6-month time points were calculated for completing participants, and using last outcome carried forward (LCF) imputation assuming data were missing at random. When screening verbal pain intensity or duration differed from recorded registration data (e.g., enrolled as “acute” with 3-month follow-up but later endorsing ongoing LBP for 5 years), registration data was used for categorization, but subjects were not re-consented to extend the study duration. A linear regression assessed interaction with any factors differing by enrollment groups.

The one-tailed significance level was set at 0.025 for noninferiority tests and two-tailed at.05 for comparison statistics, reporting 95% confidence intervals. Data analysis was performed using STATANow/SE 18.5 and MedCalc https://www.medcalc.org/calc/fisher.php (Version 23.2.1; accessed April 14, 2025).

## Results

### Participants

**Figure 2:**
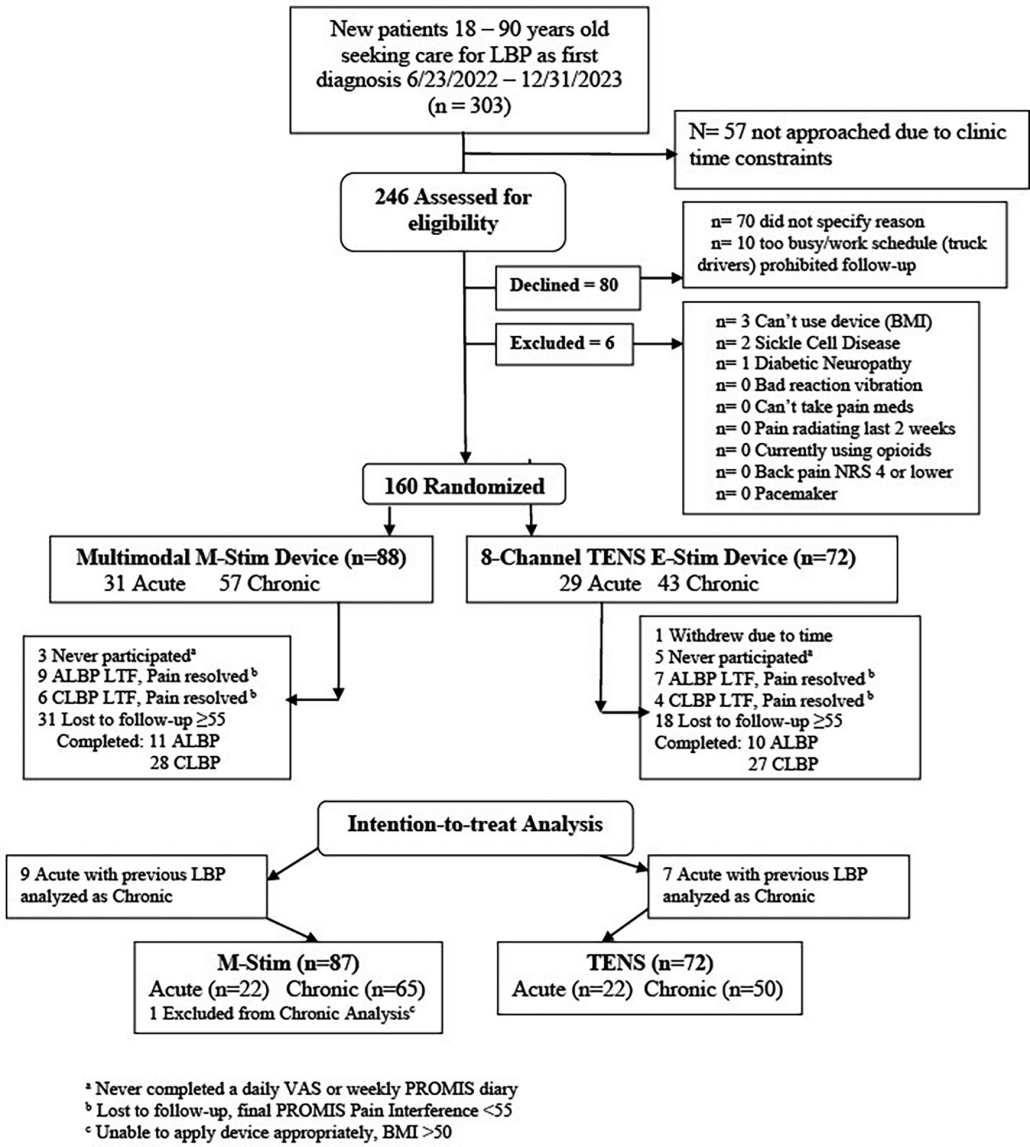
The consort flowchart acute and chronic Low back pain.

We enrolled 160 participants, of whom 159 were eligible (M-Stim = 87, TENS = 72); one enrolled M-Stim subject (BMI = 60) was unable to apply the device. (Figure 2).

The majority were female (54.7%), non-Hispanic (94.3%), and Black or Multiple Race (72.9%). The average age was 41.1 years (SD = 12.2), BMI 30.9(SD6.19) and 31% had a household income less than $75,000. Average initial pain intensity was 5.51(SD2.15) on the 0–10 NRS, with a PROMIS Pain Interference T-Score of 63.65(SD7.18), (high moderate severity); 115(72%) had chronic pain for at least 3 months, 55% used opioids for their pain, and 112 cLBP and 39 aLBP met the definition of intractable pain (unresponsive to 3 or more interventions). (Table 1) Of M-Stim participants, 74(85%) completed 28 days, similar to the TENS users 60(83%); overall 73.6% completed 3 months and 55/100 enrolled as cLBP completed 6 months. One participant per group completed no diary entries; 3 M-stim and 5 TENS participants completed no PROMIS disability entries. Diary entries by TENS users [mean 29.4(SD11.74)] reported 68.1 min and 2.02 device uses per day, while M-Stim diaries [29.3(SD10.5)] averaged 65.5 min and 2.15 device uses per day. Average diary entry and device use frequency did not differ by intervention, duration, or baseline pain.

**Table 1:** Sociodemographic and clinical characteristics.

Characteristic	Total (*n* = 159)	DuoTherm (*n* = 87)	TENS (*n* = 72)
Age, mean [SD], years	42.58 [12.31]	40.85 [11.97]	44.67 [12.47]
Female [No. (%)]	86 (54.09)	48 (55.17)	38 (52.78)
Race			
Black	106 (66.67)	60 (68.97)	46 (63.89)
White	23 (14.47)	10 (11.49)	13 (18.06)
AI/AN, Asian, or Hawaiian/PI	10 (6.29)	5 (5.75)	5 (6.94)
Unknown/Not Reported	9 (5.66)	6 (6.90)	3 (4.17)
Multiple	11 (6.92)	6 (6.90)	5 (6.94)
Ethnicity—Hispanic or Latino	12 (7.55)	9 (12.5)	3 (3.45)
Marital status			
Married/Domestic Partner	79 (49.69)	38 (43.68)	41 (56.94)
Never married	62 (38.99)	37 (42.53)	25 (34.72)
Divorced/Separated/Widowed	18 (11.32)	12 (13.79)	6 (8.33)
Highest complete education			
Less than High School	10 (6.29)	6 (6.90)	4 (5.56)
High School	48 (30.19)	24 (27.59)	24 (33.33)
Associates or Technical Degree	20 (12.58)	9 (10.34)	11 (15.28)
Baccalaureate or Higher	81 (50.94)	48 (55.18)	33 (45.84)
Household income			
Less than $75K	38 (23.9)	19 (21.84)	19 (26.39)
At least $75K	83 (52.2)	49 (56.32)	34 (47.22)
Prefer not to answer	38 (23.9)	19 (21.84)	19 (26.39)
Not employed	26 (16.35)	14 (16.09)	12 (16.67)
Average size of household	2.72 [1.42]	2.69 (1.51)	2.75 (1.31)
Substance use			
Current smoker (Y) (PCORI)	19 (11.95)	9 (10.34)	10 (13.89)
Drunk/used drugs more than you wanted (>“Never”)	33 (20.75)	13 (14.94)	20 (27.78)
Wanted/needed to cut down on drinking/drug^a^ (>“Rarely”)	13 (8.18)	6 (6.90)	7 (9.72)
Back pain history			
Duration of low back pain			
Acute (<3 m)	44 (27.67)	22 (25.29)	22 (30.56)
Chronic (≥3 m)	115 (72.33)	65 (74.71)	50 (69.44)
Chronic >5 years	62 (38.99)	34 (39.08)	28 (38.89)
History of MVC	61 (38.36)	33 (37.93)	28 (38.89)
Have filed workers’ compensation claim?	12 (7.55)	8 (9.20)	4 (5.56)
Involved in legal claim? (Y)	34 (21.38)	18 (20.69)	16 (22.22)
Filed disability for LBP?	13 (8.18)	10 (11.49)	3 (4.17)
Pain relief interventions			
Prior opioid use for back pain (Y)	80 (50.31)	44 (50.57)	36 (50.00)
Prior or current gabapentin (Y)	17 (10.69)	9 (10.34)	8 (13.89)
TENS (Y)	47 (29.56)	24 (27.59)	23 (31.94)
Vibration^a^ (Y)	60 (37.73)	27 (31.03)	33 (45.83)
Cannabis (Y)	21 (13.21)	11 (12.64)	10 (13.89)
Cognitive behavioral therapy (Y)	10 (6.29)	4 (4.60)	6 (8.33)
Exercise at least once weekly	124 (76.19)	67 (75.61)	57 (76.92)
Tried ≥5 of 11 Integrative Treatments	53 (33.33)	26 (29.89)	27 (37.5)
Surgical or procedural interventions			
Previous back operation (Y)	9 (5.66)	6 (6.90)	3 (4.17)
Spinal fusion^a^ (Y)	3 (1.89)	2 (2.30)	1 (1.39)
Back injections (Y)	29 (18.24)	18 (20.69)	11 (15.28)
Physical			
BMI^a^, Mean [SD]	30.86 [6.19]	29.88 [5.22]	**32.04 [7.05]**
BMI by category			
<25	26 (16.35)	14 (16.09)	12 (16.67)
25–29.9	55 (34.59)	34 (39.08)	21 (29.17)
≥30	78 (49.06)	39 (44.83)	39 (54.17)
Current back pain characteristics			
Pain at least half of the days (Y)	127 (79.87)	70 (80.46)	57 (79.17)
Pain Intensity Now NRS_1^b^ 0–10	5.51 [2.15]	5.53 [2.02]	5.48 [2.32]
Pain Intensity 24 h NRS_2^c^	6.39 [2.19]	6.5 [1.99]	6.25 [2.42]
PROMIS Pain Intensity^a^ (7 days worst 1–5)	4.02 [0.79]	**4.14 [0.73]**	3.88 [0.84]
PROMIS 7 days average (1–5)	3.22 [0.89]	3.22 [0.75]	3.22 [0.89]
PROMIS pain interference T-score (Normed at 50, higher worse)	63.65 [7.18]	63.56 [5.91]	63.75 [7.18]
PROMIS physical function T-score (Normed at 50, lower is worse)	36.57 [0.40]	36.32 [4.67]	36.87 [5.55]
PROMIS depression T-score	50.40 [0.77]	50.28 [1.03]	50.55 [1.18]
Catastrophizing >22 (out of 52)	21.45 [13.71]	21.51 [13.87]	21.38 [13.63]

Bold indicates the group with the number associated with worse LBP, *P* < 0.05.

^a^ *P*-value < 0.05.

^b^ Missing one score in TENS group.

^c^ Missing one score per group.

Of participants initially presenting with aLBP assigned to 3-month follow-up, 16 recorded prior low back pain in their registration dataset (acute-on-chronic) and were analyzed for change over time with cLBP participants. Baseline pain and disability in enrolled acute and chronic participants verified appropriateness of allocation, with acute-on-chronic more closely resembling chronic (Missing Data, Supplementary Material S4).

### Pain intensity outcomes

**Figure 3:**
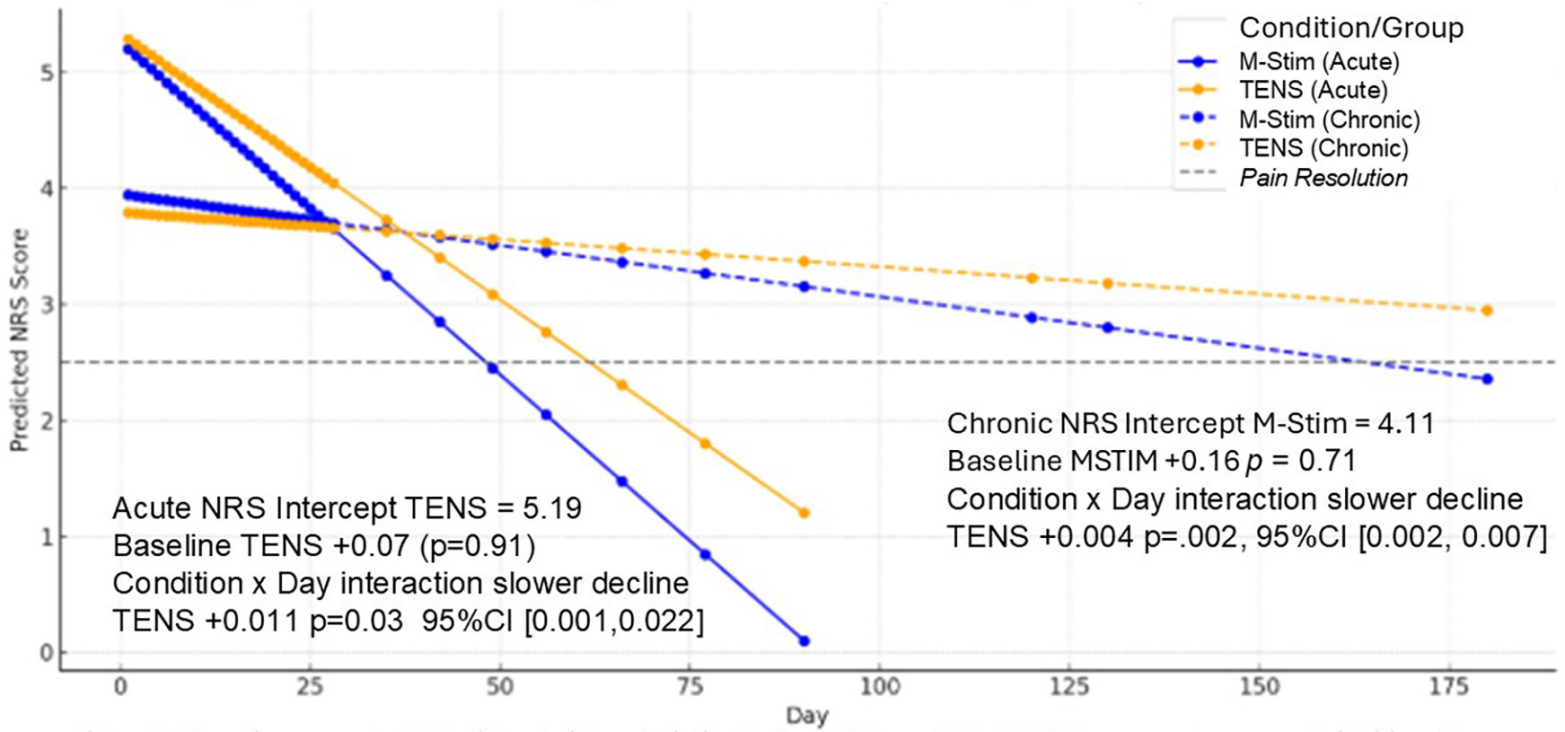
LMM change in NRS pain intensity over time. Baseline average NRS did not differ statistically for aLBP or cLBP. All NRS scores decreased significantly over the course of follow-up, with TENS decreasing less and significantly more slowly than M-Stim. All average predicted NRS endpoints showed resolution of NRS Pain Intensity except cLBP TENS subjects.

Initial 30-min and 10-day NRS acute pain relief were similar for both aLBP and cLBP, with M-Stim noninferior to TENS (aLBP 95%CI1.02,0.98, *p* < .0001, cLBP 95%CI0.58,0.72, *P* < .0001). Using Linear Mixed Model (LMM) intention-to-treat analysis for 44 aLBP and 115 cLBP participants, NRS pain intensity decreased significantly more rapidly over time for M-Stim as compared to TENS. (Figure 3) Both M-Stim and TENS aLBP participants and chronic M-Stim participants averaged final pain intensity below 2.5 (Supplementary Material S7).

### Chronic low back pain and disability outcomes

**Figure 4:**
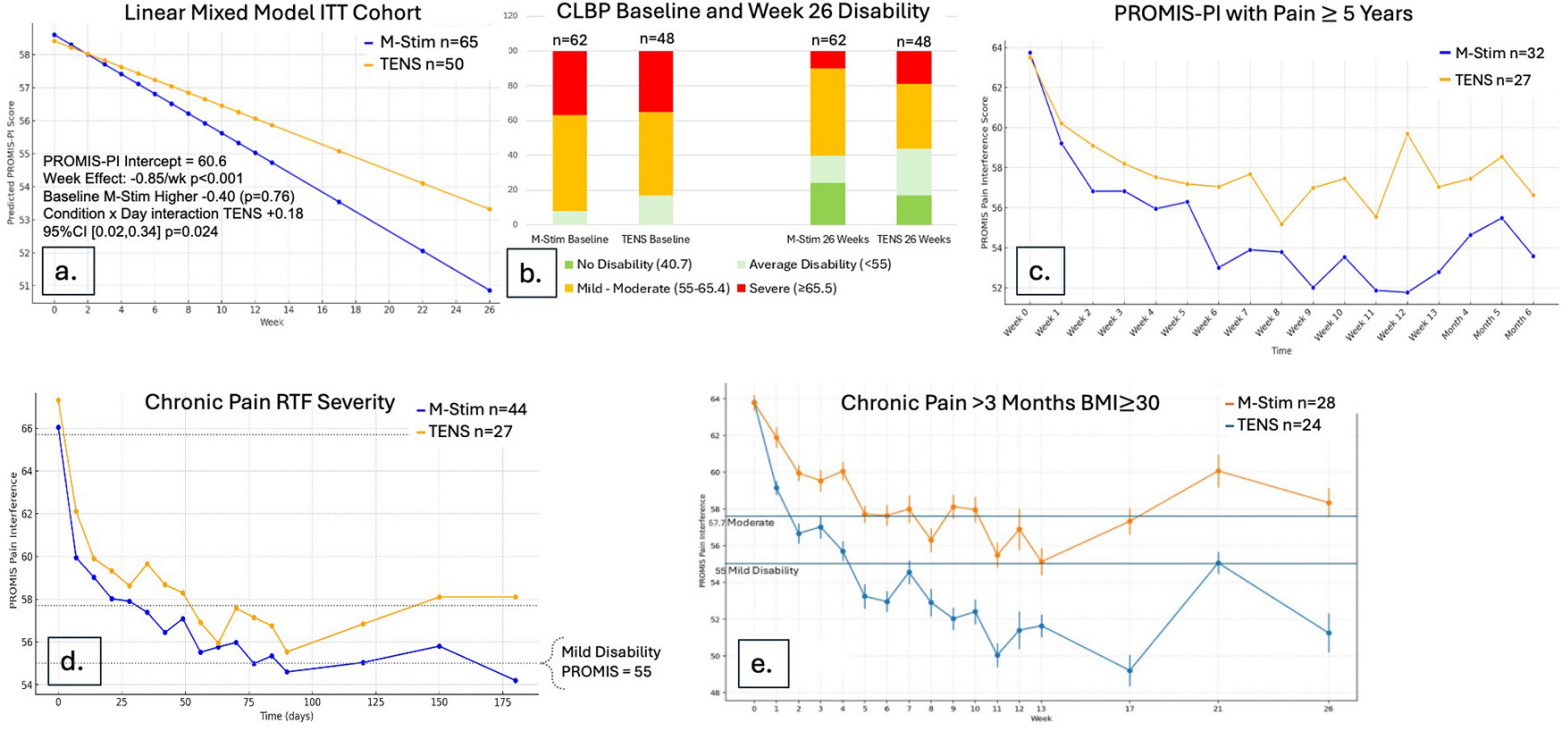
Disability reduction over time for cLBP cohort. **(a)** Using a Linear Mixed Model in ITT cohort, M-Stim regained function more rapidly. **(b)** Proportions of disability category for those completing ≥1 diary. **(c)** Mean disability scores by month for those with pain ≥5 years and completing ≥1 diary. **(d)** Disability scores RTF Moderate-to-Severe for those completing ≥1 diary. **(e)** Disability scores with 95%CI for those with BMI > 30 and completing ≥1 diary.

Using LMM intention-to-treat analysis for 115 cLBP participants, pain interference decreased significantly more rapidly over time for M-Stim (*n* = 65) as compared to TENS (*n* = 50), with a Condition × Week interaction of +0.18 (0.02, 0.34, *p* = .024) (Figure 4a).

Of those recording at least one follow-up response, M-Stim (*n* = 62) subjects had worse initial disability. (Figure 4b) Significantly more M-Stim subjects had >1 standard deviation of improvement (27/65 versus 15/50) *p* = 0.05 (Table 2). For those with pain 5 or more years, M-Stim had significantly improved Pain Interference beginning at week 10 [+4.5 (95%CI 0.28, 8.73) *p* = 0.036], with 12/32 reaching average pain compared to 4/27 using TENS [RR 0.73 (95%CI 0.54, 1) *p* = 0.051]. (Figure 4c) Significantly more M-Stim subjects meeting RTF severity criteria achieved zero disability [23.9% vs. 7.1%, RR = 0.82 (95% CI 0.68, .99) *p* = 0.042] (Figure 4d). For those with BMI >30 by ITT, M-Stim users averaged lower pain over time (Figure 4e, NS), with significantly reduced pain interference for those completing the trial (−13) versus TENS (−5.2, *p* = 0.01) (Table 2).

**Table 2:** PROMIS pain interference using LCF imputation—chronic disability.

Measure	All Chronic M-STIM	All Chronic TENS	*p*	RTF >27 Intractable M-STIM	RTF >27 Intractable TENS	*p*	BMI > 30 M-STIM	BMI > 30 TENS	*p*	Integrated Intractable M-STIM	Integrated Intractable TENS	*p*
*n*	65	50		46	28		30	26		19	18	
Baseline Mean [SD]	63.4 [6.0]	62.6 [7.0]	NS	65.9 [4.5]	67.3 [5.49]	NS	63.7 [5.2]	62.9 [6.3]	NS	65.7 [4.5]	67.6 [5.1]	NS
Week 13	53.8 [8.2]	53.6 [8.6]	NS	54.9 [8.6]	55.5 [9.3]	NS	53.1 [7.7]	55.3 [9.2]	NS	**52.5 [7.5]**	**59.0 [8.2]**	**0**.**02**
*Δ* 13 weeks	−9.6 [8.4]	−9.0[8.6]	NS	−11.1 [8.5]	−11.8 [8.5]	NS	−10.6 [8.2]	−7.6[7.7]	NS	−13.3 [8.4]	−8.6 [7.4]	NS
% change 13 weeks	−14.8%	−14.0%	NS	−16.7%	−17.5%	NS	−16.2%	−12.1%	NS	−19.9%	−12.6%	NS
Week 26 all	54.8 [8.8]	55.9 [8.5]	NS	55.2 [9.7]	59.1 [8.3]	NS	54.5 [8.3]	57.6 [8.6]	0.19	**53.4 [8.3]**	**61.1 [7.8]**	**0**.**007**
Completing	52.7 [10.4]	55.5 [8.1]	NS	54.4 [10.4]	60.2 [5.7]	0.06	**50.1 [9.5]**	**56.8 [8.3]**	**0.05**	**52.1 [8.0]**	**60.2 [7.5]**	**0**.**03**
n Completing	*n* = 27	*n* = 27		*n* = 20	*n* = 15		*n* = 14	*n* = 16		*n* = 9	*n* = 13	
*Δ* ITT	−8.5 [9.3]	−6.7 [8.3]	0.28	−10.8 [9.1]	−8.2 [7.7]	0.22	−9.3[9.1]	−5.4[7.1]	0.08	−12.2 [8.3]	**−6.5 [6.4]**	**0**.**025**
*Δ* Completing	**−10.9[9.2]**	**−6.5 [7.0]**	**0.05**	**−12.1 [9.0]**	**−5.9[5.4]**	**0.03**	**−13.0 [10.0]**	**−5.2[5.8]**	**0.01**	**−13.1 [9.0]**	**−5.4 [6.1]**	**0.03**
% change 26 weeks	−13.2%	−10.3%	NS	−16.5%	−12.3%	NS	−14.2%	−8.4%	0.1	**−18.5%**	**−9.6%**	**0**.**022**
>10-pt better at 26 weeks	**41.5% (27/65)** ^a^	**30.0% (15/50)** ^a^	**0.05** ^a^	50.0% (23/46)	35.7% (10/28)	NS	40.0% (12/30)	23.1% (6/26)	NS	52.6% (10/19)	27.8% (5/18)	0.17
>20-pt better at 26 weeks	16.9% (11/65)	6.0% (3/50)	0.06	**23.9% (11/46)** ^b^	**7.1% (2/28)** ^b^	**0.04** ^b^	16.7% (5/30)	3.8% (1/26)	0.1	26.3% (5/19)	5.6% (1/18)	0.1
% PROMIS <55	38.5% (25/65)^k^	42.0% (21/50)	0.7	41.3% (19/46)	21.4% (6/28)	0.07	36.7% (11/30)	30.8% (8/26)	NS	**47.4% (9/19)** ^c^	**11.1% (2/18)** ^c^	**0**.**025**^c^
No disability PROMIS = 40.7	23.1% (15/65)	16.0% (8/50)	0.3	**23.9% (11/46)** ^b^	**7.1% (2/28)** ^b^	**0.04** ^b^	23.3% (7/30)	11.5% (3/26)	NS	**21.1% (4/19)**	**5.6% (1/18)** ^d^	**0**.**016**^d^

Bold indicates statistically significant differences.

^a^ RR = 0.73 [95% CI 0.53–1.00] *p* = 0.05.

^b^ RR = 0.82 [95% CI 0.68–0.99] *p* = 0.04.

^c^ RR = 0.59 [95% CI 0.38–0.93] *p* = 0.025.

^d^ RR = 0.75 [95% CI 0.60–0.95] *p* = 0.016.

### Integrated analysis with common criteria

After initiation of the study, research connecting cLBP to dysfunction of the multifidus and erector spinae muscles (6, 55, 56) suggested a plausible mechanism by which multimodal M-Stim impacted function. To explore this hypothesis, we conducted an integrated analysis of intractable inoperable mechanical cLBP in the initial (31) and 1 year follow-up (51) studies of implanted multifidus E-Stim device studies (ReActiv8, Mainstay Medical, Dublin, Ireland, $27,000), using common inclusion and exclusion criteria. (Table 3).

**Table 3:** Common characteristics for multifidus intervention integrated analysis.

Intervention	M-Stim (*n* = 19)	TENS (*n* = 18)	Reactiv8 (*n* = 204)	Reactiv8 (*n* = 53)
Age	42.7 [13]	49.2 [11]	47 [9]	44 [10]
Male/Female	6 (32%)-13 (68%)	4 (22%)-14 (78%)	94 (46%)-110 (54%)	23 (43%)-30 (57%)
Opioid use	63%	77%	37%	72%
BMI	30.4 [5.1]	34.5 [7.4]	28 [4]	Not reported
Fusion/surgery	5 (26%)	2 (9%)	25 (12%)	Excluded
Epidural	6 (32%)	3 (17%)	99 (49%)	Not reported
Baseline	65.7 [4.46]	67.6 [5.05]	65 [3.43]	66.6 [3.04]
PROMIS	ODI 39.2	ODI 44 [10]	ODI 39 [10.3]	ODI 44 [10]
Month 3	52.5 [7.5]	59.0 [8.24]	n/a	61.36 [0.8) ODI 30.6 [2.2]
Month 6	53.4 [7.02]	61.1 [7.77]	58.33 [0.3] ODI 22.7 [1]	62.38 ODI 32.4 [2.4]
1 year	n/a	n/a	57.8 ODI 20.7(1)	n/a

Disability values were converted using the slope of each interval to assign a PROMIS Pain Interference estimate based on the reported mean ODI at each timepoint, where Moderate Disability: 21–40 ODI = 57.7–65.4 PROMIS PI, Severe Disability: ODI 41–60 = 65.7–71.5 (50).

M-Stim restored normal function for 47.4% by week 13 [Mean 52.5 (7.5)], with improvement persisting and significant at week 26 [47.4% resolution v 11.1%, RR = 0.59 (95% CI 0.38–0.93) *p* = 0.025] (Table 2). Final Pain Interference was in the normal range for M-Stim [53.4 (8.5)] and moderate for TENS [61.1 (7.8)] (Supplementary Material S7).

### Acute disability and progression to chronic pain

**Figure 5:**
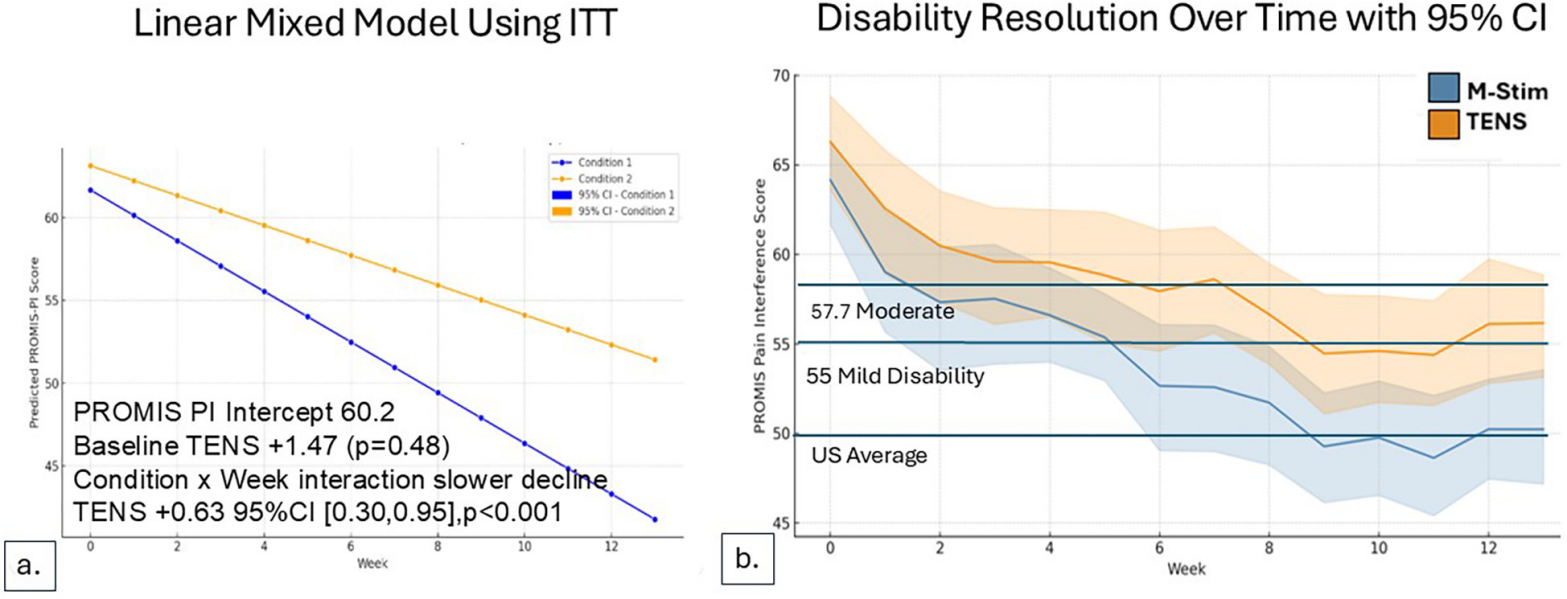
Disability reduction over time for acute pain cohort. For those with LBP duration <3 months, Pain Interference Disability Scores decreased over time. **(a)** LMM model using intention to treat, M-Stim users had more rapid resolution of disability. **(b)** Reduction with weekly SD for aLBP with ≥1 diary.

**Figure 6:**
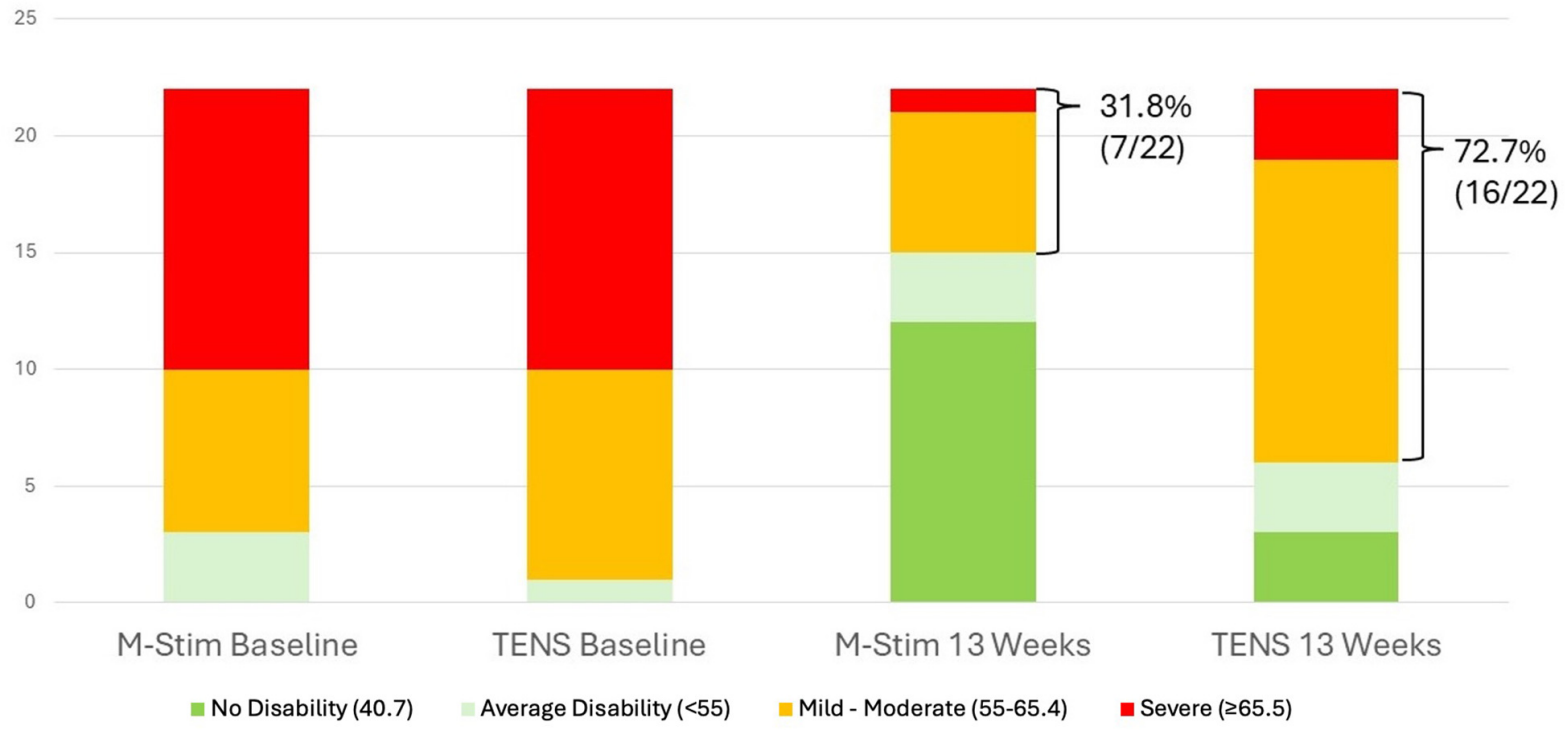
Acute pain baseline vs. Week 13 Disability. The relative risk of persistent pain at 13 weeks was significantly lower in the M-Stim group [RR = 0.44 (95% CI 0.23–0.85) *p* = 0.015, NNT = 2.4].

For the 22 aLBP participants in each intervention group, 16 listed motor vehicle collisions as the etiology of pain. Disability improved significantly more rapidly for aLBP M-Stim users, with a LMM condition × week interaction for TENS +0.63 95%CI [0.30, 0.95], *p* < 0.001. (Figure 5) Defining progression as persistent Pain Interference of mild or greater (≥55–77) at 13 weeks after enrollment, significantly fewer aLBP M-Stim users (7/22) than those using TENS (16/22) transitioned to cLBP. [RR = 0.44 (95% CI 0.23–0.85) *p* = .015, NNT = 2.4]. (Figure 6) In the RTF severity subset, M-Stim users were significantly more likely to report complete resolution of disability (T-Score = 40.7) at 13 weeks than TENS users (Table 4).

**Table 4:** Acute PROMIS pain interference using LCF imputation with ≥1 diary entry.

Measure	All acute M-STIM	All acute TENS	RTF >27 (mean 35.9) M-STIM	RTF >27 (mean 38.2) TENS
*n*	22	19	18	15
Baseline mean (SD)	64.2 [6.0]	66.6 [7.0]	66.5 [2.4]	69.4 [4.6]
Week 13	48.9 [8.21]	53.6 [8.64]	50.2 [10.2]	54.7 [8.9]
*Δ* 13 weeks	−15.26 [8.37]	−12.4 [8.63]	−16.3 [10.0]	−14.8 [8.3]
>5-pt better (13w)	72.7% (16/22)	73.7% (14/19)	72.2% (13/18)	86.7% (13/15)
>10-pt better (13w)	59% (13/22)	63.2% (12/19)	61.1% (11/18)	73.3% (11/15)
>20-pt better (13w)	45.5% (10/22)	26.3% (5/19)	50.0% (9/18)	33.3% (5/15)
%<55 at 13w No LCF 2m+	**68.2% (15/22)** ^a^ **91.6% (11/12)**	**31.6% (6/19)** ^a^ **33% (4/12)**	61.1% (11/18)	33.3% (5/15)
% 40.7 no disability No LCF 2m+	**54.5% (12/22)** ^b^ **75% (9/12)**	**15.8% (3/19)** ^b^ **25% (3/12)**	**50% (9/18)** ^c^	**13% (2/15)** ^c^

^a^ Likelihood of average to normal disability at 13 weeks RR 0.47 [95% CI 0.23 to 0.92, NNT 2.7] *p* = 0.028.

^b^ Likelihood of zero disability at 13 weeks RR 0.54 [95% CI 0.32 to 0.89, NNT 2.58] *p* = 0.015.

^c^ Likelihood of zero disability at 13 weeks RR 0.58 [95% CI 0.35 to 0.95, NNT 2.72] *p* = 0.032.

### Neural network and device use

**Figure 7:**
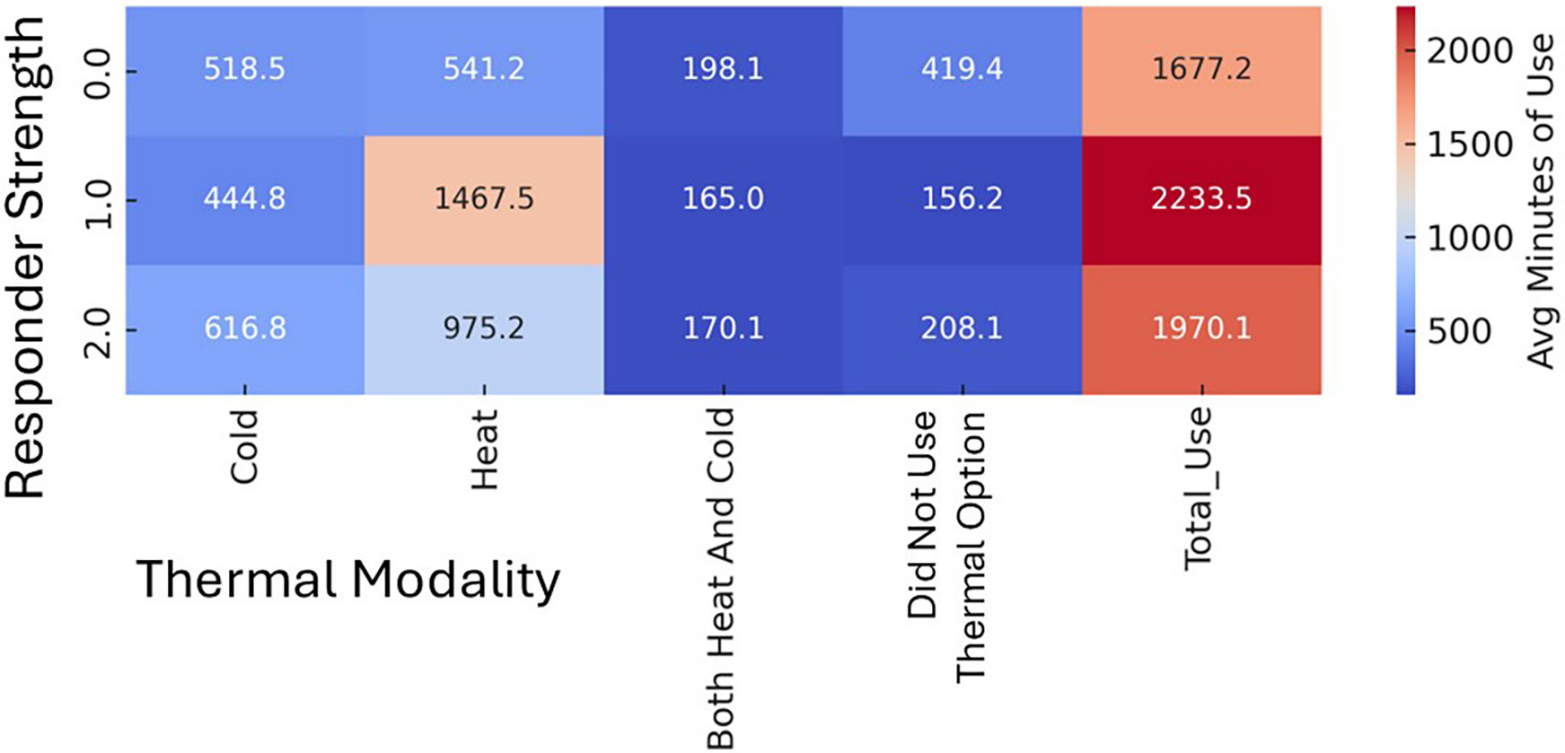
Thermal Use patterns in chronic participants by responder Status. Strong Responder (2.0) = zero (PROMIS = 40.7) disability **AND** >10-pt Improvement **OR** t-score below Moderate Disability **AND** >20-pt Improvement **OR** initial t-score Severe **AND** >15-pt Improvement. Mild Responder (1.0) has PROMIS ≤50 **AND** >5-pt improvements **OR** less than Moderate Disability **AND** >10-pt improvement OR initial Severe **AND** >10-pt Improvement. All others (0.0) are non-responders.

Features most strongly associated with improved function were a history of motor vehicle collision, thermal therapy application concurrent with device (only available with M-Stim), and greater device usage frequency. Factors associated with reduced improvement included higher BMI, prior opioid use for LBP, and higher initial pain scores. Model performance was evaluated using mean absolute error and R², demonstrating improved predictive accuracy over baseline. (Supplementary Material S6) Average device time per use and use per day did not differ between groups or responders within groups. In the M-Stim group, responders tended to use the device more walking or working than in bed (*p* = .11), while non-responders were less likely to use any thermal interventions (Figure 7).

### Blinding assessment and opioid use

The most common initial response in both groups was to answer the question regarding the study purpose without answering the allocation question “do you think you were in the treatment or control group?”. Considering this combination an “I don’t know” response, allocation guesses were made by 56 in the M-Stim group (8 “Control”, 29 Don't Know, 19 “Treatment”) and 49 using TENS (12 “Control”, 26 Don't Know, 11 “Treatment”). Using Bang's Blinding Index, M-Stim BBI = −.032 (95%CI −0.54, −0.06) and TENS BBI = −0.51 (95%CI −0.71, −0.24), both significantly against their allocation groups. Using the James Index, M-Stim = 0.54, TENS = 0.71, where values greater than 0.5 indicate adequate blinding.

First 28-day opioid use was reduced in the M-Stim group by 44.6% (32.33 milligram morphine equivalents, *p* = 0.02), but increased in the TENS group. There were no differences in other analgesic use, and no reported adverse events.

## Discussion

In those seeking treatment at a chiropractic office for moderate-to-severe LBP, disability improved significantly more rapidly and to a greater extent for participants allocated to M-Stim for acute or chronic pain. ALBP participants assigned to the M-Stim device were significantly less likely to transition to cLBP. M-Stim's improvement of function was significant for participants with greater severity and cLBP duration, with the strongest results for the subset of intractable inoperable mechanical cLBP who had failed other interventions. This study supports the importance of multimodal options for pain management and suggests a novel role of harmonic M-Stim frequencies to restore function for those suffering from chronic mechanical LBP.

LBP is the leading cause of disability worldwide (57) and directly contributes $200 billion in annual US healthcare costs (58–61). As the most common reason for ambulatory opioid prescribing in the US (62), LBP was a logical target for the NIH Help End Addiction LongTerm (HEAL) initiative: 5% of those prescribed opioids become chronic users (63), and averting one case of OUD is estimated to save US taxpayers $325,125 (64). Current LBP guidelines support first-line multimodal interventions rather than opioids (65, 66), but physician pain management education focuses on pharmaceuticals, not integrative options (67). This study verifies that M-Stim reduces acute LBP pain at least as well as TENS. The establishment of a single FDA-approved multimodal pain device at point-of-care could promote options other than opioid prescribing.

Early physical exercise, heat, acupuncture and physiotherapy prevent cLBP progression (17, 23, 68), but are difficult to implement rapidly. In a 76-clinic US initiative to enhance early intervention, progression of moderate-to-severe aLBP to cLBP was unchanged at 40% (2). DuoTherm combines multiple elements of these preventative modalities: somatosensory heat and pressure for central and neural effects, with mechanical force-based frequencies associated with vasodilation, reduced muscle damage, and neuromotor reflex improvement for tissue effects. If the findings for acute pain are replicated, early initiation could have a significant impact for back injuries. In 2022 the 67,510 low back pain injuries in transportation workers cost an average $39,000 and 9 days off work (72); at $5,000, the M-Stim intervention would save over $2.2B per year. As LBP affects 60% of Americans each year (1), preventing 13% from progressing to cLBP could help 7.8 million people at a savings of $2,000 per patient per year in direct costs (73).

M-Stim and TENS were similar in efficacy for acute pain intensity, but intensity is less predictive of opioid use and pain chronicity than lack of coping strategies (74). In addition to central and local tissue effects, having multiple options when initial pain relief fades enhances self-efficacy and reduces pain catastrophizing (44, 69–71). The contribution of different relief modalities, different stochastic patterns of mechanical force emphasizing different frequencies, and thermal variations to self-efficacy vs. cellular mechanisms should be explored in future work.

Effective M-Stim mechanisms may differ for cLBP. The efficacy of implanted muscular rather than spinal cord stimulation for these patients supports that ongoing nociceptive cLBP requires paraspinal muscle rehabilitation rather than neuromodulatory or inflammatory blockade. The (75) outsized role of fascia in nociception and inflammation, and the discovery of reparative cellular proteins activated by mechanical force are all now active areas of investigation. Multimodal M-Stim was more likely to improve function in severely impacted cLPB subsets. Given the myriad effects of focal vibration (repeated mechanical force) recently described in the literature, hypotheses include direct analgesia (27), neuromuscular recovery (29), and low back pain specific effects (25) through targeting paraspinal muscles and fascia.

### Paraspinal muscles and mechanical Low back pain

For 90% of LBP patients, imaging shows no nerve or bony target for intervention (14). Systematic reviews of the spine-stabilizing multifidus and erector spinae muscles (6, 55, 56), their overlying fascia (5, 10, 19), and the role of neuromotor control now support a common pathway to ongoing LBP initially hypothesized in 2007 by Langevin et al. (76) Muscle injury causes local inflammation, with cold reducing pain transmission and cytokine release (77, 78). Stabilizing muscles are recruited, with resultant edema and increased metabolic requirements exceeding local blood supply. Pain from the resulting ischemia and ongoing spasm (79) may respond to heat at this phase. Within a week of bracing or voluntary immobility due to pain, fatty infiltration of the muscles is seen (8), further reducing blood flow and increasing pain from ceramide production (7). Altered neuromotor responses develop rapidly in the presence of inflammation (3), leading to maladaptive motor control contributing to reinjury (80, 81) Ongoing relative ischemia and dysfunction of the multifidus lead to adherence of the fascia (10) disproportionately increasing pain with movement (19), causing a disordered feedback loop: plastic changes of 1a afferent proprioception muscle spindles and supraspinal motor coordination reflexes (82, 83) lead to spinal instability and neuromotor reflex dysfunction (84). Central conditioning and fear reduce movements (85), compounding the effect of immobilization, but can be reduced with early movement and exercise (73). These neuromotor changes with aLBP predict cLBP (4), manifesting as poor proprioceptive control and stability (82, 86). Thus the superiority of multimodal over opioid treatments can be explained: opioid lethargy may reduce movement leading to increased pain, while early anti-inflammatory medications and cold followed by heat and exercise mitigate the physiologic progression of mechanical nociceptive processes.

### Cellular discoveries: mechanical force ion channels

While pain and inflammatory processes have been extensively described, the role of mechanical force in cellular adaptation has only been recognized in the last decades. Ion channels typically depolarize with electric and chemical activity. Piezo1 channels are activated directly by pressure and held open for ion influx with proteins using this force (e.g. Yoda1, Jedi1) (87). Effects include musculoskeletal growth, vascular effects, and specific channels related to pain (87, 88). These channels are reversibly opened, so intermittent pressure, i.e. vibration can repeatedly activate cell activity (109), including specifically fatty lipid remodeling (89).

### Vibration for pain, vascular and myofascial rehabilitation

M-Stim simultaneously employs vibratory effects described in different disciplines to achieve varied effects. Vibration for nociception and itch was first well described by Wall in 1960, predating his more famous 1965 gate control theory (90). In the early 1990s, Salter identified the neurotransmitters and optimal frequency to block nociception. Pacinian fast-touch mechanoreceptors transmit vibration impulses directly to the dorsal horn, rather than joining wide dynamic range neurons with less specific nociception inhibition (10, 24, 25, 27, 91–93). Pacinian ATP released optimally at 200 Hz becomes adenosine, the purine responsible for presynaptic inhibition (24, 94). Single-motor 200 Hz devices have been found effective in over 100 studies for sharp nociceptive pain, with greater efficacy when placed in multiple dermatomes (27, 95).

This short-term neuromodulatory effect alone cannot explain the chronic improvement in LBP. Lundeberg's vibrating back plates reduced pain better with 200 Hz than 100 Hz, with duration-dependent sustained relief (36, 96). Since Lundeberg's time, vibration in this range has demonstrated varied tissue effects associated with repair. For example, frequencies in the 150 Hz range increase blood flow and wound healing via nitric oxide release (97, 98). Potentially, applying either single frequency across a field over time addressed hypoperfusion in the multifidus and erector spinae. Some frequencies of vibration directly inhibit fatty muscular changes, and may reverse them over time (7, 39, 99). Multiple frequencies increase range of motion through tissue changes (100–102); specific frequencies reduce inflammatory calcitonin gene-related peptide (40). Inhibitory pain relief relies on oxytocin (103–105), which vibration increases, and a growing dataset from the field of kinesiology supports vibration to directly reconfigure neuromotor derangement and fascial and muscle dysfunction (29, 106). By using multiple motors, stochastic constructive interference arises in the areas between nodes, giving exponentially greater variety in amplitude and nodal locations to address multiple physiologic derangements in a field. Therapeutic synergies may therefore arise not just from thermomechanical combinations, but specific combinatory mechanical stimulation patterns.

### Pain and physics-based energy modalities

That biology-based nutrients and chemical compounds have specialized effects on the human body is axiomatic: indications for vitamin C vs. vitamin B12, or antacids vs. antidepressants differ to the point of malpractice if misprescribed. Our findings support a similar specificity for evolving physics-based modalities. Just as biochemical therapeutics must match receptor targets and physiologic pathways, administration of energy via electricity, force, light and radiation must match the physiology, mechanism, and mechanical properties of the target tissues.

Current occurs when charged particles flow through the least electrically-resistant tissue between positive and negative electrodes. Electrical stimulation external to tissue generates electrical and magnetic fields. Periodic mechanical force from sound or vibration propagates as pressure waves, while kinetic energy causes cellular and tissue shear or deformation. Thermal energy passes via changed molecular energy, while light energy is absorbed, scattered, and refracted depending on wavelength (116, 117).

While charge is the currency of biologic function (118), not all functions generate or respond to current. Pain incorporates biologic, chemical, and electrophysiologic events. Noxious temperature, pressure, or chemical exposures trigger nociception via cation and ligand-gated protein channels. These peripheral pain signals propagate centrally via voltage-gated sodium channels, the phylogenetically simplest and fastest method to conduct depolarization. Once the voltage-mediated message reaches the central cell bodies, released pain neurotransmitters activate transduction and response through synaptic mechanisms. Activated and blocked neurotransmitter receptors change cell functions, using cations through complex protein channels as second messengers or to depolarize intracellularly (107).

Therapeutic physics interventions depend not only on the targets’ mechanism of action, but mechanical factors inherent in the tissues. Piezo1 channels are activated by mechanical shear, pressure, or stretch deformation. Typically calcium then flows intracellularly to act as a second messenger to initiate cascades of cellular activity (108). Repeated delivery of kinetic energy may be beneficial up to a cellular tolerance, accounting for specific vibration frequencies in different tissues. (109). Vectors, frequency and amplitude must match tissue tolerance and desired outcomes.

### Study findings relating to energy and tissue targets

The high number of MVC participants may have contributed to M-Stim's greater impact on disability, particularly for the aLBP progression. Fascial adherence or paraspinal fatty changes from immobilization, whether through bracing or pain relief, are more common after trauma than overuse. Vibratory effects of vasodilation and inhibiting fatty changes may better address this type of derangement. The trend toward better outcomes with movement over bed-use may support the contention in the multifidus literature supporting neuromotor reflex repair (32). The kinesiology literature suggests vibration with active muscle contraction is preferred to reset dysfunctional reflexes from pain and asynchronous proprioception (29). Interestingly, the “30 minutes 3× a day for 3 days” recommended by Fattorini et al. was frequently the use pattern for strong responders. Future studies evaluating acute-to-chronic transition should evaluate the contribution of activity, stretching, or passive use in conjunction with vibration to outcomes. In addition, using big data to correlate etiology, thermal use, duration, and the most chosen therapy cycles are areas for future investigation.

A striking finding was the comparison of two non-invasive interventions against paraspinal muscle stimulator surgery. As studies with that intervention lacked a control, our data could provide some insight using the common criteria. However, while spine research supports PROMIS Pain Interference indicators of disability as superior to both ODI and Likert Scales (110, 111), the bell curve distribution makes a 1:1 crosswalk estimation more difficult. To bias toward the null hypothesis, we used the more lenient crosswalk for moderate scores (ODI 20 = PI 57.7) from the LBP literature rather than the general PROMIS PI score cut-point of 60. As the sample size for our participants was both much smaller and followed for less time, these results must be interpreted with caution.

While all participants in the DuoTherm pilot study endorsed “would recommend”, this study did not ask participants to rate satisfaction with the devices. Changes in pain catastrophizing would have been interesting to know, but this information was not collected. Future research evaluating enjoyment or greater feelings of agency with the devices would be helpful to ascertain these contributions to the effectiveness of M-Stim.

### Limitations

In the context of LPB research broadly, this study has numerous strengths, including subject BMI and socioeconomic status that better represent US national averages than typical surgical or post-op studies of similar severity. The use of an active control allowed for successful blinding, which is extremely rare in device studies. Moreover, the 6-month follow-up illuminated comparisons beyond the time expected for reversion to the mean or placebo effects. Daily collection of not just prescribed but prior and external opioid sources covered a full month, while many opioid LPB studies only follow use for prescriptions given at the point of care and at a weekly or monthly cadence. With regards to limitations, the choice of a chiropractic office to avoid prescribing bias could reduce generalizability, although the percent using opioids (51%) was similar to other non-chiropractic studies (2). As both early physical care and spinal manipulation are associated with improved relief, the rapidity of pain resolution we found in both groups may differ from other outpatient environments. While MVC injuries are more likely to lead to chronic pain (112), the preponderance these participants might introduce bias in favor of M-Stim, as vibration and thermal interventions are particularly well-suited for mechanical pain.

Because cLBP research reports PROMIS and disability measures to be more relevant than NRS, we used “mild disability” as described in PROMIS guidelines as our cut-off for ongoing cLBP (110, 111). While the difference in transition from acute to chronic pain was statistically significant, the percent of TENS patients who still had mild pain at 3 months (72%) was higher than previously reported. However, any more stringent criteria would have also reduced the percentage of M-Stim participants without ongoing 3-month pain. The sample size of true acute patients should be replicated with larger numbers, and more detailed descriptions of etiology (2, 73, 113).

While the primary LMM analysis using intention to treat was significant for both acute pain and disability in both chronicity groups, analyses for smaller subsets may have been underpowered. Because the LMM analysis and subset results ubiquitously favored M-Stim, to emphasize responder signal we felt it was appropriate not to apply a Bonferroni correction. Relative risks for chronic subsets should be considered with this decision in mind. Contrariwise, the broad socioeconomic inclusion criteria may have artificially reduced statistical power: subjects with ongoing lawsuits, high pain catastrophizing and depression are typically excluded by E-Stim multifidus and other surgical intervention studies to avoid blunting the significance of physiologic interventions.

Finally, the superiority of M-Stim in those with higher BMI may reflect a reduced penetration of TENS rather than superior M-Stim efficacy.

## Conclusion

In comparison to prescription TENS, adding M-Stim devices significantly reduced the transition of acute to chronic LBP, and restored function significantly more in subjects with higher severity. The combination of multiple therapies addressing the physiology of acute, intermediate, and chronic injury, particularly with stochastic harmonic interaction of specific focal vibration frequencies in a novel array, may be potentially useful to address the growing epidemic of low back pain (63, 114, 115).

## Data Availability

The raw data supporting the conclusions of this article will be made available by the authors, without undue reservation.
