# Supplementary material for: Novel multimodal mechanical stimulation is superior to TENS to treat and prevent chronic low back pain: a randomized controlled trial
Source: Front Pain Res (Lausanne). 2025 Aug 18;6:1625420. doi: 10.3389/fpain.2025.1625420 (PMC12399623; doi:10.3389/fpain.2025.1625420)
Supplement: Supplementary file 1 [file Datasheet1.docx]

Supplemental Material:

|  |  | **Pg.** |
| --- | --- | --- |
| **1** | SPIRIT Table for M-Stim Low Back Pain and Opioid Prevention | **2** |
| **2** | DOSE: Dose Opioid Source Evaluation Tool | **9** |
| **3** | Informed Consent and Data Collection Instrument | **12** |
| **4** | Missing Data Plan | **39** |
| **5** | Data Sharing Statement | **48** |

**6** Neural Network 13-Week Prediction Model **49**

**7** Supplemental Data (PROMIS and NRS) **53**

**8** References **57**

**Supplement 1: SPIRIT Table: eProtocol for M-Stim for Low Back Pain and Opioid Prescribing**

| Administrative | Description |
| --- | --- |
| Title | Multimodal Mechanical Stimulation to Reduce Opioid Prescribing for Low Back Pain: A Randomized Controlled Trial;  Multimodal Mechanical Stimulation to Prevent Chronic Low Back Pain and Treat Intractable Disability: A Randomized Controlled Trial |
| Trial Registration | ClinicalTrials.gov NCT04491175 |
| Protocol Version | NIH funded protocol submitted 5/29/2019 to FDA Q191165  IRB Protocol Submission Date: 8/10/2019  Protocol Amendment Number: 4  Authors: A.B.,K.S.,L.L.  Revision Chronology:   1. 8/10/2019 Original   [COVID delay]   1. 4/21/2022 FDA recommended modifications from Q191165 incorporated to protocol: increased sample size to 60 opioid naïve and 100 chronic (with 6 months followup); Replaced Sham with LG Smart TENS and added a blinding assessment; Outcome measures of PROMIS Pain Interference , Pain Intensity, Depression to be measured weekly the first month. 2. 6/8/2022 – Final survey design reduced daily diary reporting to 1 month due to budget constraints of longer followup. Depression and Catastrophizing only as baseline; weekly NRS Pain Interference (3) PROMIS Pain Interference (2) and Pain Intensity collected until 3 months for all; Monthly surveys added past month NRS, Blinding assessment for additional 3 months for those started in chronic strata.   Added “source” for each opioid use case to DOSE tool collecting opioid formulation, dose, and number of pills.   1. 5/5/23; Added “Do you think you will need surgery” and “do you think you will need epidural” questions, and “did you get surgery” and “did you get an epidural” to monthly. 2. 7/13/2023 New Location added |
| Funding | National Institute on Drug Abuse, R44DA049631, R44DA058952 |
| Roles and Responsibilities | Principal Investigator: Amy Baxter MD, responsible for study design, overall project oversight, and compliance. Study Coordinator: Jena Slaski, responsible for daily operation, protocol adherence and patient coordination at Kaizo Health Centers. Lindsey Cohen assisted Dr. Baxter with initial study design, ongoing recruitment recommendations, pain measure decision-making, study flow creation. M. Louise Lawson PhD (Deceased), responsible for initial power analysis, statistical support for study design, revisions after FDA. Dr. Kevin Swartout PhD, responsible for programming of data collection instruments, ongoing support of data collection tool and dataset, database consolidation; Jessica Allia Williams PhD, responsible for statistical analysis of completed dataset. All apart from Dr. Lawson reviewed the manuscript. |
| **Introduction** | Low back pain (LBP) affects nearly 60% of adults yearly, with 40% progressing from acute to chronic pain. It is the single biggest contributor to worldwide disability. The opioid epidemic, largely driven by prescriptions for pain, has created an urgent need for effective non-pharmacological pain treatments. Existing options such as TENS devices may provide acute relief, but are not covered as an opioid substitute. A multimodal approach, combining mechanical stimulation, heat, cold, and acupressure, holds promise for reducing both acute and chronic pain and opioid prescribing. |
| Background and rationale for the study | Between 25 and 50% of patients presenting with acute or exacerbated chronic LBP (a/cLBP) receive prescriptions for opioids. ALBP progresses to chronic in 40% of those with moderate-to-severe pain (4 out of 10 on a 0 – 10 Numeric Rating Scale). Opioid pain relief is no better than placebo at 6 weeks after presentation, but increases the risk of cLBP and persistent use (5% of opioid naïve). Providing a multimodal low back pain device could reduce opioid prescribing or use compared to TENS; providing any device could reduce opioid prescribing and use compared to contemporaneous prescribing practices, leading to a new FDA indication if 50% relief or 30% improvement compared to an active control. |
| Objectives | **Primary Objective, Opioid Reduction:** To determine whether the use of a multimodal neuromodulatory mechanical stimulation (M-Stim) device (DuoTherm) can reduce prescribing of opioid medications 30% compared to TENS in the opioid-naive with moderate to severe back pain. **Secondary Objectives:** To determine whether multimodal M-Stim reduces the days of use and use after 7 days in the opioid-naïve, and versus contemporaneous national prescribing, and using MME for those with chronic opioid use for LBP.  **Primary Objective, CLBP Reduction:** To determine whether M-Stim reduces disability compared to TENS over 3 or 6 months using PROMIS Pain Interference. Secondary Objectives: To determine whether M-Stim is noninferior compared to TENS for acute pain using a NRS after 30 minutes and 10 days; to compare NRS after 3 and 6 months; to evaluate the progression of acute to chronic pain over 3 months for those with aLBP. |
| Study Design | A randomized, double-blind controlled trial with 3 m followup for opioid outcomes, and 3-aLPB or 6- month (cLPB) follow-up for pain outcomes. |
| Study Setting | The study will be conducted at mixed physical therapy/chiropractic practices in Washington, DC, Maryland, and Virginia, which offer demographic and economic diversity. These clinics provide an ideal environment, with frequent follow-ups and support for alternative therapies but no on-site access to opioid prescribers. |
| Eligibility Criteria | Inclusion Criteria: - Male or female adults aged 18-90 with diagnosed acute or chronic low back pain  - Self report moderate to severe LBP of 4 or greater out of 10 on a 0-10 Numeric Rating Scale.  - Acute: pain duration <3 months without opioid use for low back pain  - Chronic: Pain duration >=3 months with or without chronic opioid use - Capacity to understand risks and benefits (informed consent).  - Smartphone for Qualtrics web-based platform use Exclusion Criteria:  - Radicular pain likely reflecting a surgical or mechanical problem  - Inability to apply or use the device, BMI (>30 with prototype, >50 with new device) or staff evaluation of fit with appropriate use (sitting or standing)  - Sensitivity to cold or vibration (e.g Raynaud’s or Sickle Cell Disease)  - Diabetic neuropathy rendering a patient unable to determine if the device is too hot  - New neurologic deficits, skin lesions over the low back area  - A contraindication to any medication for pain management that would impact analgesic use record  - Pacemaker |
| Intervention | The experimental arm is a novel multimodal low back pain relief device incorporating 8 harmonic vibration patterns of mechanical stimulation (M-Stim), and optional heat, cold, and pressure delivered through a sculpted metal plate attached with a belt and controlled by buttons on the belt. The active control is a prescription 4-lead 8-channel TENS unit (LG Smart). Participants will be instructed to use the device for a minimum of 30 minutes a day. Both M-Stim and LG Smart TENS groups will receive a standard pain management regimen typically used for low back pain standardized across all clinicians participating in the study. Participants will keep the devices and be instructed to use them for pain as needed during the duration of the study. |
| Outcomes | The **Opioid** primary study question was whether a multimodal pain relief device reduces opioid prescribing and use compared to TENS when added to other treatments for those with outpatient moderate-to-severe pain reduce. We hypothesized that providing a multimodal M-Stim device would reduce prescribing by 30% in the opioid naïve. Secondary outcomes were MME (Milligram Morphine Equivalents) between the first 14 days and the last 14 days of daily diary recordings for those who took opioids, and reduced opioid prescribing compared to national rates compared to TENS.  **Secondary outcomes** included risk factors of prolonged opioid use (POU) in the opioid naive, including days of use, continued opioid use after 7 days, and total MME in the opioid naïve, and prescribing compared to a contemporaneous national LBP population (25%).  The **Pain and Disability** primary study questions were whether a multimodal device reduced immediate and 10-day Pain Intensity using a numeric rating scale (NRS) 30% better than TENS (acute), and whether it reduced cLBP disability better than TENS using PROMIS Pain Interference at 6 months. |
| Participant Timeline | \|  \| Initial Visit \| Daily x 28 days \| Weekly for 3 months \| Monthly for 3 months \| Monthly 4 - 6 months \| \| --- \| --- \| --- \| --- \| --- \| --- \| \| Consent, Enrollment, Randomization \| Albp, clbp \|  \|  \|  \|  \| \| Minimum Dataset, Pain Catastrophizing Sullivan, PROMIS Depression, Physical Function \| Albp, clbp \|  \|  \|  \|  \| \| NRS (at that moment & past 24 hours) \| Albp, clbp \| ALBP, CLBP \| Albp, clbp \| Albp, clbp \| clbp \| \| NRS after 30 minutes \| Albp, clbp \|  \|  \|  \|  \| \| NRS Past week \|  \|  \| Albp, clbp \| Albp, clbp \| clbp \| \| NRS Past month \|  \|  \|  \| Albp, clbp \| CLBP \| \| PROMIS Pain Intensity, Pain Interference \| Albp, clbp \|  \| Albp, clbp \| Albp, clbp \| CLBP \| \| Dose, Opioid formulation, number Source (DOSE tool), other analgesics \| Albp, clbp \| ALBP, CLBP \| ALBP, CLBP \| ALBP, CLBP \| CLBP \| \| Device usage \|  \| ALBP, CLBP \| ALBP, CLBP \| ALBP, CLBP \| CLBP \| |
| Sample Size | To estimate the M-Stim intervention effect size, a retrospective study of 59 cLBP patients on chronic opioids (100% prolonged opioid use) reported a 28% reduction in MME after spinal stimulator implantation,[1] reducing MME 26.2(SD32.8) compared to 5.8(SD34.2) for those with conventional treatment (effect size 0.60. A priori, we would be able to detect an effect size of 1 (meaning a difference of 1 standard deviation) with 94% power and alpha of .03 using a Wilcoxon Mann Whitney test with 30 in each opioid-naïve group. With this effect size for a two-sided significance of 0.05, 23 participants per group would detect a 30% prescribing reduction. For cLBP, 50 participants on per treatment group would provide 85.4% power to detect a 30% reduction in MME. We planned to recruit 60 participants who had never taken opioids for their back pain, and 100 cLBP for disability outcomes with or without chronic opioid use for cLBP. |
| Recruitment | Patients were recruited from Kaizo Health Centers in Landover and Fairfax, through direct referrals, flyers, and digital content like social posts and emails targeting local populations. Flyers were also distributed at three local pain clinics. |
| Randomization | After eligibility screening and informed consent, a 4-digit participant ID and the intervention allocation were [randomly assigned](https://www.qualtrics.com/support/survey-platform/survey-module/survey-flow/standard-elements/randomizer/)  by the survey software to either the DuoTherm or LG Smart TENS group within acute and chronic arms. A copy of the informed consent was emailed to the participant. |
| Blinding (masking) | Participants were blinded to which device powered the study hypothesis, and blinded to their assigned intervention until after documenting initial NRS pain intensity. Success of blinding the study device (M-Stim) versus the active control (TENS) was tested at months 3-6 with prompts, “select which group you think you were in (control or treatment)” and “How confident are you (0 = Least Confident ... 100 = Most Confident).”  The protocol statistician and study coordinator knew the intervention assignments during enrollment but did not conduct statistical analysis. The PI and treating chiropractor were blinded to allocation during enrollment, with the PI accessing data only after study completion. The analyzing statistician was blinded to device assignment until completion of data cleaning and initial analysis. |
| Data Collection Methods | Site coordinators entered identification and contact information after informed consent on a tablet, then recorded weight, height, and duration to confirm acute or chronic follow-up duration, then participants took the tablet to record pain intensity on a 0-10 NRS now and past 24 hours. While using the device for 30 minutes, participants completed 7-day pain intensity NRS, NIH Back Pain Research Minimum Dataset, including PROMIS measures of Physical Function, Pain Interference, Depression, and Catastrophizing via the Sullivan Catastrophizing scale, Injury Mechanism, prior treatments, exercise and athletic status.  Participants were prompted to record analgesic use, pain, and device use daily for a month on the web-based platform, then weekly, with the monthly surveys adding Pain Interference. All surveys asked about analgesic use. Due to the anticipated heterogeneity of opioid formulations, a skip-logic data collection instrument algorithm was created with robust options for Dose per pill (34), Opioid formulations, e.g. hydrocodone, hydromorphone etc. (15) and the Source (DOSE tool, Supplement 2). Sources included “prescribed to me for this event”, “prescribed to me for another event”, “given to me by a family member”, “given to me by a friend”, “given to me by an acquaintance or stranger”, or “purchased from someone without a prescription”. These were converted to a total milligram morphine equivalent (MME) for analysis.  Participants recorded when they used their device, duration of use, and any additional pain relief modalities (exercise, stretching, bath, hot tub, therapy, massage). Patients were asked to record any thermal use or therapy cycle or channel as appropriate to their device.  Participants verbally endorsing pain for less than three months (acute) were entered into a 3 month follow-up program, or 6 months (chronic) for those for whom back pain had been an ongoing problem for more than 3 months. |
| Statistical Methods | Statistical analysis was performed with masked intervention allocation by a statistician new to the project to determine differences in primary and secondary outcomes.  An intention-to-treat analysis included all participants meeting inclusion criteria. Summary statistics (means, standard deviations, proportions) were calculated using T-tests and Chi-squared tests. **Opioid** prescribing differences were calculated with raw percent of change and relative risks. Diary entries and item-level data were analyzed for missing data to assess the need for imputation. Relative risks were calculated for MME and opioid use days between intervention groups, with paired T-tests used to compare first two and last two weeks of MME in those who used opioids during the study. Prescribing was followed for 3 months. A linear regression assessed BMI's interaction with opioid use, calculating marginal effects with standard errors via the Delta Method.  Pain outcomes will be analyzed using intention-to-treat, imputed using Last Observation Carried Forward. Disability outcomes use Healthmeasures definition of mild disability = 55. For comparison to ODI, numeric estimates of PROMIS Pain Interference scores were derived by linearly interpolating published ODI-to-PROMIS crosswalk by Tang et al,[2] where an ODI of 20–40 (moderate) corresponds to PROMIS scores of 57.7–65.4 and an ODI of 41–60 (severe) corresponds to PROMIS scores of 65.7–71.5. Values were converted using the slope of each interval to assign a PROMIS estimate based on the reported mean ODI at each timepoint. Data analysis was performed using STATANow/SE 18.5 and online MedCalc. |
| Data Monitoring | Data collection was conducted through an app that tracked daily pain, opioid use and device usage. Monitoring included weekly enrollment review by the protocol statistician to assess comprehension of the data collection tool. Adverse events were reported as per protocol.  Obtained data was stored on password-protected network drives made accessible solely to the listed investigators. Data analysis will only be conducted in secure rooms with restricted access. These rooms will only be accessible to investigators. Patient samples will not be obtained. The Institutional Review Board reserves the right to audit any research files to assure the quality of any data used in this research |
| Safety/Harm | Q191165/S001 FDA ruled Non-significant Risk 18-Sept 2019. Risks are minimal, similar to those associated with a handheld massager or standard heat/cold therapy. Patients will be screened for sensitivity to cold or vibration. Any adverse events will be reported to the IRB and NIH within 24 hours. |
| Auditing | Periodic data auditing will be conducted by the Institutional Review Board (IRB) to ensure compliance with protocol and safety regulations, and by site visits by the PI. |
| Ethics Approval | This trial has received ethics approval from the Kaizo Clinical Research Institute IRB and is registered under NCT04491175. |
| Protocol Amendments | Revision Chronology:   1. 8/10/2019 Original   [COVID delay]   1. 4/21/2022 Incorporated FDA recommended modifications from Q191165 incorporated to protocol: increased sample size to 60 opioid naïve acute and 100 chronic (with 6 months followup); Replaced Sham with LG Smart TENS active control with 8 pre-programmed patterns to correspond with 8 pre-set DuoTherm therapy cycles and added blinding assessment. Outcome measures of PROMIS Pain Interference , Pain Intensity, Depression to be measured weekly the first month. We extended the follow-up from one week for acute pain to 3 months for acute pain and 6 months for chronic pain. 2. 6/8/2022 – Final survey design reduced daily diary reporting to 1 month due to budget constraints of longer follow-up. Depression and Catastrophizing only as baseline; weekly NRS Pain Interference (3) PROMIS Pain Interference (2) and Pain Intensity collected until 3 months for all; “Past month NRS” added to monthly surveys added past month NRS, Blinding assessment for additional 3 months for those started in chronic strata.   Added “source” for each opioid use case to DOSE tool collecting opioid formulation, dose, and number of pills.  We intended to use evaluate change in MME over time for chronic opioid users. The FDA recommended requiring participants on opioids maintain dosage and frequency. As we did not have access to prescribing records, and opioid reduction was a desired outcome measure, we evaluated opioid use for the first two weeks versus the last two of those endorsing prior opioid use. We extended the baseline and follow-up Pain Intensity measurements to one month rather than one week.   1. 5/5/23; Added “Do you think you will need surgery” and “do you think you will need epidural” questions, and “did you get surgery” and “did you get an epidural” to monthly. 2. 7/13/2023 New Location added   **Stratification by chronicity versus opioid use status:** enrollment strata were based on verbal endorsement of duration and no acute opioid use for back pain. Initial evaluation of registration data after enrollment was complete revealed 16 of 60 in the “acute” strata reported a duration longer than three months, and half in each chronicity strata no prior opioids for LBP. In consultation with Clinical Trials.gov, we pooled the data and maintained the acute/opioid naïve registry for opioid use outcomes NCT04491175, and chronic/mixed for pain outcomes NCT04494698. |
| Informed Consent | Informed consent will be obtained from each participant after screening. Participants will be informed about the voluntary nature of the study, with a HIPAA form for confidentiality. Consent will be documented via selecting ‘continue’ on the digital consent form. |
| Confidentiality | Patient data will be stored on password-protected network drives. All data will be de-identified using a unique study ID for each participant. Confidential records will be stored in secure, restricted-access rooms. |
| Declaration of interests | The principal investigator has financial conflict of interest from design and research into the DuoTherm device, and creating a company to develop the device with NIH SBIR funding. The collaborators declare no financial or personal conflicts of interest related to this study. |
| Dissemination Policy | Results will be disseminated through peer-reviewed journals, conferences, and possibly through NIH publications, ensuring that both positive and negative outcomes are reported. |
| Ancillary and Post-Trial Care | Patients will receive standard care post-trial and may keep their devices if found beneficial. Participants will be provided referrals for ongoing care as needed. |
| Publication Plan | High impact journal, with presentation at the IASP or similar pain-related conference. |

**Supplement 2: DOSE Tool**

Due to the heterogeneity of potential outpatient opioid formulations, a data collection instrument algorithm was created to collect Dose, Opioid, and the Source of different brands in circulation without relying on free text. Using skip-logic prompts programmed into Qualtrics (Provo, Utah, USA), during registration participants were asked “What short acting opioid medications have you taken for your pain” and with daily and weekly surveys “…since your last survey? (Please select all that apply)”.

The potential choices included 8 opioid formulations (e.g. Hydrocodone/APAP (acetaminophen)) followed by all potential brands currently on the market (e.g. “Hydrocodone/APAP (acetaminophen) [ex: Lorcet, Lortab, Norco, Vicodin, Zydone, Generic]) or “I did not take any short-acting opioids”. The next prompt asked which specific brand was taken, giving choices of all dose combinations for that brand and how many pills were taken. The final prompt of “This medication was …” probed for source with 6 possible choices: “prescribed to me for this event”, “prescribed to me for another event”, “given to me by a family member”, “given to me by a friend”, “given to me by an acquaintance or stranger”, or “purchased from someone without a prescription”. The same question was asked for six long-acting opioids. Opioid prescribing outcomes included any response by any participant that included “to me for this event”, while opioid use included any recorded opioids. To compare all quantitative opioid responses, MME were calculated using the Heath and Human Services conversion tables.[3]


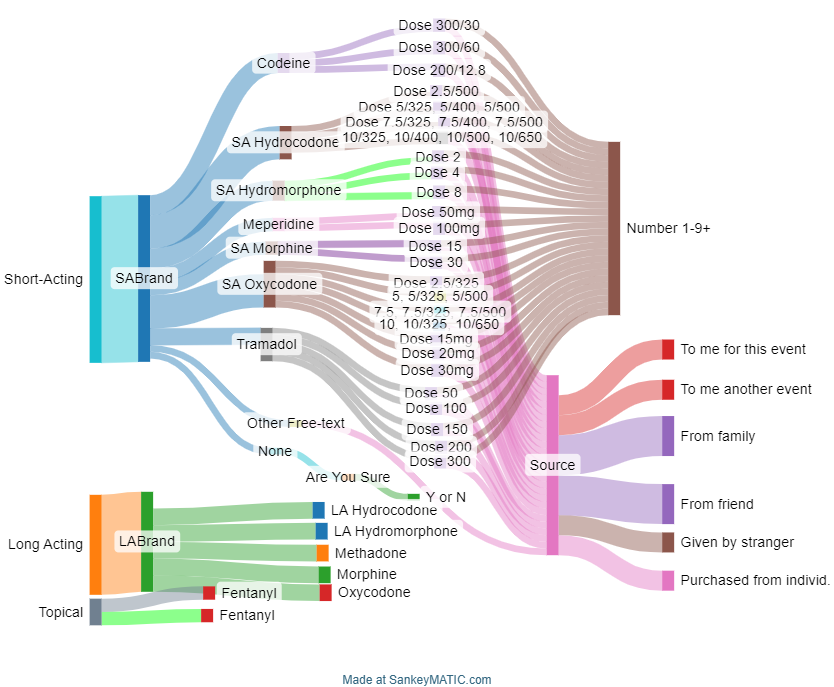


The initial DOSE tool will be modified in future iterations based on study outcomes.

The category of “Other Short Acting [ex: Dihydromorphine, Nicomorphine, Oxycodone with Aspirin or Ibuprofen, Oxymorphone]” will be removed and the answers will not be considered in the opioid use, as the subjects appeared to be responding to the Aspirin or Ibuprofen prompt, not realizing it was a combination product.

**The next iteration of the tool will allow for overwriting when backspaces are applied.**

Fentanyl patch use or medication assisted treatment (buprenorphine).

**Future iterations will not allow more than 1 week recordings and will allow for free-texting an amount if more than 9 pills.**

When an opioid has both a long and a short acting form, and the participant entered the same name, dose, pill number and source under both SA and LA, **future iterations will include logic to verify and eliminate one or the other.**

Supplement 3: Informed Consent and Data Collection

Registration Survey (Acute)

Start of Block: Consent

**Participant Registration Survey (Pain <3m)**

Which location is enrolling the patient in the study?

- Fairfax, VA
- Landover, MD

**MOVE TO THE NEXT PAGE
 THEN HAND TABLET TO PARTICIPANT**

[Page Break]

**INFORMED CONSENT FORM**
    
**Addressing Opioid Use Disorder with an External Multimodal Neuromodulation Device: Clinical Evaluation for Opioid-Sparing in Acute Low Back Pain**

 You have been invited to take part in this study. It is important that you read and understand the info below before you agree to participate. Your relationship with the clinic will not change if you decide not to be in the study. Please ask questions about what you do not understand before agreeing to take part.

 **Purpose of the Study**
 The purpose of this study is to determine whether providing a stimulation device to patients with acute low back pain (LBP) reduces pain and opioid use.

**Procedures**
 If you agree to be in this study, we will ask you to do the following things: After completing the initial paperwork, you will rate your pain and complete background paperwork. Next, the computer program will determine your group assignment to one of two neuromodulation devices designed for daily home use using either mechanical or electrical stimulation both with multiple therapy cycles.

 After your group assignment, you will receive a standard pain management regimen typically used for low back pain. Regardless of group assignment, you will be instructed on how to use the data collection system via an online instruction video. We are collecting information on opioid use. Therefore, we will ask you to complete the surveys on how much medication you are taking, including if there are different kinds that may be from different sources and medication you have taken prior to getting a prescription. You will be prompted by the **text message with a link** to record the following information on the website: Daily for one month – use of pain medication and pain relief (Device, exercise, stretching, bath, hot tub, therapy, massage, etc.). Weekly – how pain interferes with movement and how intense it is Months 1, 2, 3– changes in function and your mood Your participation will consist of an hour for paperwork/instruction today and then a quick 2-minutes survey daily for a month. Each week there will be a 15-minute questionnaire for the full three months of the study.

[Enrollment Chart from SPIRIT Table]

***Risks* and *Benefits* of being in the study**

 **Possible Risks:** The risks of the device should be no greater than the risks of a hand-held massager, electrical stimulator, or hot or cold pack depending on which group you’re in. The possibility exists that the focus on pain assessment will draw attention to the LBP and could increase your pain. You will be given your own device so risk of transmitting infection will be minimal. In previous research, patients with LBP supported that the use of the device is helpful; no patient has stated that it increases pain or causes other problems. The risk of receiving no intervention is the same risk which is standard of care for patients being treated for LBP throughout the country. Electrical stimulation is already a standard of care and found safe for LBP. Cold and heat are already a standard of care in home remedies for LBP – the possibility of increased sensitivity to cold exists, but you choose whether or not to use the cold portion if you’re in this group. Patients receiving cold therapy will not be exposed to enough cold to result in frostbite. Likewise, if you do not want heat, you do not have to use heat with your device, or if you do not want electricity you don’t need to use it.

 **Benefits:** This study will determine whether different kinds of low back stimulation for patients with LBP will reduce opioid use. Achieving our aims implies, you may reduce your use of pain medication or not need to start opioids. The results of the study may also be translated to other musculoskeletal complaints where inflammation, pain and stiffness are concerns that decrease quality of life. If the stimulation device increases compliance with medical care, it may diminish pain, stress and reduce opioid use, which provides a significant benefit to you. Conceptually it will challenge the pharmaceutical focus of current pain strategies, changing practice in the field of pain relief and rehabilitation. If there are lower unused opioids in circulation, this is also an important addition to combatting opioid use disorder.

**Confidentiality**
 All info you reveal in this study will be kept confidential. Your records will be assigned a random number instead of using your name. Documents will be stored in a locked cabinet and electronic files will be kept on a secure computer. Only members of the research team will have access to these records. Three years after the study, we will shred all papers. When the study is published your name will not be included. Your records may be looked at by the Kaizo Clinical Research Institute IRB, Department of Health and Human Development, the Food and Drug Administration, and state and federal agencies.

 **Compensation**
 You will be compensated for each of the questionnaires you complete according to the following schedule. Weeks 1 and 2: $1 a day for every time you complete the daily form. If you complete 7 days in a row you will earn a $3 bonus for hitting the streak. Weeks 3 and 4: $2 a day for every time you complete the daily form. If you complete 7 days in a row you will earn a $6 bonus for hitting the streak. Months 2 and 3: $10 every time you complete a weekly survey. If you complete four weekly surveys in a row you will earn a $10 bonus for hitting the streak. This will total a maximum of $160 you can earn. You will be paid with an Amazon gift card at the end of each month. You must complete 80% of the data in weeks 1 and 2 to continue to weeks 3 and 4. If you complete 80% of the data in the first month you get to keep the assigned device. If you do not, we will ask for you to return the device and you will forfeit the money earned during this first month.

**Injury or Illness**
 If you say YES to participate, then your consent in this document does not waive any of your legal rights. However, in the event you are injured or become ill as a result of participating in this study, neither Kaizo Health, Kaizo Clinical Research Institute, Kaizo Clinical Research Institute IRB, or any other researchers are able to give you any money, insurance coverage, free medical care, or any other compensation from such injury. In the event that you suffer injury as a result of participation in any research project, you may contact Dr. Jay Greenstein at (301) 518-1006 or Dr. Barton Bishop the current IRB chair at (240) 766-0300 x835 at Kaizo Clinical Research Institute, who will be glad to review the matter with you.

 **Voluntary Nature of the Study:**
 Taking part in this study is completely voluntary. You may withdraw and stop participating at any time. If at any time, you wish to withdraw from the study please let any member of the research staff know. 

 **Contacts and Questions**
 If you have any questions, you can call Jena Slaski (240) 766-0300x838. If you have concerns about your rights as a research participant, you can contact Kaizo Clinical Research Institute IRB Chair, Barton Bishop at (240) 766-0300x835.

 **Statement of Consent**

 **I have read the info above. I have had the chance to ask questions and have them answered. Tapping “next” below signifies that I agree to participate in the study and have been given a copy of the consent form.**

[Page Break]

**PLEASE HAND THE TABLET TO THE STAFF MEMBER.**

End of Block: Consent

Start of Block: Registration Survey

**PLEASE RECORD THE FOLLOWING INFORMATION IN THE PARTICIPANT'S FILE**

Participant ID:**${e://Field/LoginID}**

Display This Question:

If Condition = 1

Participant Condition:  **DT**

Display This Question:

If Condition = 2

Participant Condition:  **LG**

[Page Break]

Participant Contact Information and Body Measurements (completed by staff member)

- First Name __________________________________________________
- Last Name __________________________________________________
- Email Address __________________________________________________
- Cell phone number (1-555-555-5555) __________________________________________________
- Patient Height (in inches) __________________________________________________
- Patient Weight (in lbs) __________________________________________________

How long has the patient experienced ongoing back pain? (If greater than 3 months, switch to the chronic survey.)

- 1-2 days
- 3-4 days
- 5 days-1 week
- 1-2 weeks
- 3 weeks-1 month
- 1-2 months
- 2-3 months

[Page Break]
**MOVE TO THE NEXT PAGE THEN HAND TABLET TO PARTICIPANT**

[Page Break]

| 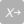 |
| --- |

Please Rate Your Current and Recent Pain Using the Sliding Scales Below   (0 = No Pain......10 = Worst Possible Pain)

|  | 0 | 1 | 2 | 3 | 4 | 5 | 6 | 7 | 8 | 9 | 10 |
| --- | --- | --- | --- | --- | --- | --- | --- | --- | --- | --- | --- |
| Pain Now |  |  |  |  |  |  |  |  |  |  |  |
| Pain over the past 24 hours |  |  |  |  |  |  |  |  |  |  |  |

At this time, how likely do you think it is you will need surgery for your low back pain issue?

- Extremely unlikely
- Unlikely
- Neither likely or unlikely
- Likely
- Extremely likely

At this time, how likely do you think it is you will need an epidural injection for your low back pain issue?

- Extremely unlikely
- Unlikely
- Neither likely or unlikely
- Likely
- Extremely likely

[Page Break]

**PLEASE HAND THE TABLET TO THE STAFF MEMBER.**
 **THEY WILL SHOW YOU THE INSTRUCTIONAL VIDEO**
**THEN BEGIN THE DEVICE THERAPY**

**Condition 1:** Instructions: <https://www.youtube.com/watch?v=BI0Bhf0ejEc>

**Condition 2**: Instructions https://www.youtube.com/watch?v=IgeURjDAgRw

[Page Break]

Time device therapy started

________________________________________________________________

[Page Break]

**MOVE TO THE NEXT PAGE THEN HAND TABLET TO PARTICIPANT**

[Page Break]

How long has low back pain been an ongoing problem for you?

- Less than 1 month
- 1-3 months
- 3-6 months
- 6 months-1 year
- 1-5 years
- More than 5 years

How often has low back pain been an ongoing problem for you over the past 3 months?

- Every day or nearly every day in the past 3 months
- At least half the days in the past 3 months
- Fewer than half the days in the past 3 months

How many days ago did your current episode of low back pain start?

- 1-2 days
- 3-4 days
- 5-6 days
- 1 week
- 2 weeks
- More than 2 weeks

Is your low back pain more severe than pain in other parts of your body?

- Yes
- No
- Not sure

How do you believe your back pain started? (select all that apply)

- Muscle pull
- Sitting/strain
- Bone injury
- Lifting
- Fall
- Work overuse
- Penetrating injury
- Motor vehicle collision
- Other (Please specify) __________________________________________________
- I don't know

| In the past 7 days… | Had no pain | | Mild | | Moderate | | Severe | | Very severe | |
| --- | --- | --- | --- | --- | --- | --- | --- | --- | --- | --- |
| How intense was your pain at its worst? |  | |  | |  | |  | |  | |
| How intense was your average pain? |  | |  | |  | |  | |  | |
| In the past 7 days... | | Not at all | | A little bit | | Somewhat | | Quite a bit | | Very much |
| How much did pain interfere with your day-to-day activities? | |  | |  | |  | |  | |  |
| How much did pain interfere with work around the home? | |  | |  | |  | |  | |  |
| How much did pain interfere with your ability to participate in social activities? | |  | |  | |  | |  | |  |
| How much did pain interfere with your household chores? | |  | |  | |  | |  | |  |
| How much did pain interfere with the things you usually do for fun? | |  | |  | |  | |  | |  |
| How much did pain interfere eith your enjoyment of social activities? | |  | |  | |  | |  | |  |
| How much did pain interfere with your enjoyment of life? | |  | |  | |  | |  | |  |
| How much did pain interfere with your family life? | |  | |  | |  | |  | |  |

Physical Function - How much difficulty do you experience with the following activities?

|  | No difficulty | A little | Some difficulty | Much | Unable to do |
| --- | --- | --- | --- | --- | --- |
| Chores such as vacuuming or yard work |  |  |  |  |  |
| Going up and down stairs at a normal pace |  |  |  |  |  |
| Go for a walk of at least 15 minutes |  |  |  |  |  |
| Run errands and shop |  |  |  |  |  |

| When I'm in pain... | Not at all | Slightly | Moderately | To a great degree | All the time |
| --- | --- | --- | --- | --- | --- |
| I worry all the time about whether the pain will end |  |  |  |  |  |
| I feel I can't go on |  |  |  |  |  |
| It's terrible and I think it's never going to get better |  |  |  |  |  |
| It's awful and I feel that it overwhelms me |  |  |  |  |  |
| I feel I can't stand it anymore |  |  |  |  |  |
| I become afraid that the pain will get worse |  |  |  |  |  |
| I keep thinking of other painful events |  |  |  |  |  |
| I anxiously want the pain to go away |  |  |  |  |  |
| I can't seem to keep it out of my mind |  |  |  |  |  |
| I keep thinking of how much it hurts |  |  |  |  |  |
| I keep thinking of how badly I want the pain to stop |  |  |  |  |  |
| There's nothing I can do to reduce the intensity of the pain |  |  |  |  |  |
| I wonder whether something serious may happen |  |  |  |  |  |

In the past 7 days...

|  | Never | Rarely | Sometimes | Often | Always |
| --- | --- | --- | --- | --- | --- |
| I felt worthless |  |  |  |  |  |
| I felt helpless |  |  |  |  |  |
| I felt depressed |  |  |  |  |  |
| I felt hopeless |  |  |  |  |  |

Have you ever had a low back operation?

- Yes, once
- Yes, more than once
- No

Display This Question:

If Have you ever had a low back operation? = Yes, once

Or Have you ever had a low back operation? = Yes, more than once

Did any of your back operations involve a spinal fusion?

- Yes
- No
- Not sure

Have you been off work or unemployed for 1 month or more due to low-back pain?

- Yes
- No
- Does not apply

Have you filed or been awarded a worker’s compensation claim related to your back problem?

- Yes
- No
- Does not apply

Are you involved in a lawsuit or legal claim related to your back problem?

- Yes
- No
- Not sure

Have you ever applied for, or received, disability insurance for your pain condition?

- Yes
- No

[Page Break]

Have you used any of the following treatment for your low back pain? (check all that apply)

- Over the counter medications
- Exercise therapy
- Injections (facet, steroid)
- Vibration therapy
- Cannabis/Marijuana
- Cold therapy
- Heat therapy
- Acupressure therapy
- Massage therapy
- TENS unit therapy
- Psychological counseling, such as Cognitive Behavioral Therapy
- Topical creams (CBD, menthol, etc.) please specify __________________________________________________
- I have not used any of these treatments

[Page Break]

How often do you exercise?

- No exercise
- 1-2 times/week
- 3+ times/week
- Competitive athlete

Display This Question:

If How often do you exercise? = 1-2 times/week

Or How often do you exercise? = 3+ times/week

Or How often do you exercise? = Competitive athlete

How vigorously do you exercise?

- Low impact (walking)
- Moderate impact
- Intense

[Page Break]

How would you describe your cigaratte smoking?

- Never smoked
- Current smoker
- Used to smoke, but have now quit

In the past year...

|  | Never | Rarely | Sometimes | Often | Always |
| --- | --- | --- | --- | --- | --- |
| Have you been drunk or used drugs more than you meant to? |  |  |  |  |  |
| Have you felt you wanted or needed to cut down on your drinking or drug use? |  |  |  |  |  |

[Page Break]

What short acting medications have you taken for your pain in the past? (Please select all that apply)

- Codeine [ex: Tylenol w/ codeine, Neurofen, T#3]
- Hydrocodone/APAP (acetaminophen) [ex: Lorcet, Lortab, Norco, Vicodin, Zydone, Generic]
- Hydromorphone [ex: Dilaudid, Palladone]
- Meperidine (Demerol)
- Short Acting Morphine Sulfates
- Oxycodone [ex: Percocet, Endocet, Tylox, Oxaydo, Roxicodone, Generic]
- Tramadol [ex: Ultram, ConZip]
- Other Short Acting [ex: Dihydromorphine, Nicomorphine, Oxycodone with Aspirin or Ibuprofen, Oxymorphone]
- I have not taken any of these short-acting medications in the past.

What long acting medications have you taken for your pain in the past? (Please select all that apply)

- Hydrocodone [ex: Hysingla, Vantrela, Zohydro]
- Hydromorphone SR [ex: Exalgo, Hydromorph Contin, Jurnista, Palladone SR]
- Methadone [ex: Amidone, Dolophine, Methadose]
- Long Acting Morphine Sulfates [ex: Avinza, MS Contin, Kadian, Oramorph SR]
- Oxycodone HCl [ex: Oxaydo, Oxycontin, Xtampza]
- Fentanyl Patch [ex: Duragesic]
- I have not taken any of these long-acting medications in the past

In the past, have you used any of the following medications for pain? (Please select all that apply)

- Acetaminophen (e.g., Tylenol)
- Gabapentin
- Ibuprofen (e.g., Advil or Motrin)
- Asprin
- Cannabis/Marijuana
- Naproxin Sodium (e.g., Aleeve)
- Another medication not listed on this page (Please specify) __________________________________________________
- I have not taken any of these medications in the past

[Page Break]

Please answer the following demographic questions about yourself.

Date of Birth

________________________________________________________________

Age

________________________________________________________________

Sex at Birth

- Male
- Female
- Unknown
- Intersex

Gender Identity

- Male
- Female
- Unknown
- Other, Please Specify __________________________________________________

Ethnicity

- Hispanic or Latino
- Not Hispanic or Latino
- Unknown
- Not Reported

Race (choose all that apply)

- American Indian or Alaskan Native
- Asian
- Black or African-American
- Native Hawaiian or Other Pacific Islander
- White
- Unknown
- Not Reported

What is the highest level of education you have completed?

- Did not complete Secondary School or Less than High School
- Some Secondary School or High School Education
- High School or Secondary School Degree Complete
- Associate’s or Technical Degree Complete
- College or Baccalaureate Degree Complete
- Doctoral or Postgraduate Education

What is your current employment status?

- Full-time employment
- Not employed
- Part-time Employment

What category best describes your current relationship status?

- Divorced
- Married
- Never married
- Separated
- Widowed
- Domestic Partner

Including yourself, how many people live in your household?

________________________________________________________________

What is your annual household income from all sources?

- Less than $10,000
- $10,000--- $24,999
- $25,000--- $34,999
- $35,000--- $49,999
- $50,000--- $74,999
- $75,000---$99,999
- $100,000--- $149,999
- $150,000--- $199,999
- $200,000 or more
- Prefer not to answer

[Page Break]

**PLEASE HAND THE TABLET BACK TO THE STAFF MEMBER**

[Page Break]

Time device therapy stopped

________________________________________________________________

[Page Break]

**MOVE TO THE NEXT PAGE
 THEN HAND TABLET TO THE PARTICIPANT**

[Page Break]

| 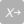 |
| --- |

**How is your pain now that you have used the study device?   (0 = No Pain......10 = Worst Possible Pain)**

|  | 0 | 1 | 2 | 3 | 4 | 5 | 6 | 7 | 8 | 9 | 10 |
| --- | --- | --- | --- | --- | --- | --- | --- | --- | --- | --- | --- |
| 1 |  |  |  |  |  |  |  |  |  |  |  |

[Page Break]

**PLEASE HAND THE TABLET BACK TO THE STAFF MEMBER**

[Page Break]

Initial visit therapy after use of study device:

- Exercises
- Adjustment
- IASTM
- Manual Therapy
- No Other Therapy

End of Block: Registration Survey

Start of Block: Post-Survey Script

**Post-Registration Script**
 Thank you for registering for this study!

 Once I record your survey responses by clicking the button below, you will receive an email with the full consent form you read at the beginning of the survey. 

 As a reminder, this is a 3-month study. You will receive emails and text messages each day for the first 4 weeks, beginning tomorrow evening, with links to complete daily surveys. These surveys are short and will not take much of your time at all. Your surveys at the end of each week (starting on your 7th day in the study) will be a little bit longer, so we wanted to give you a heads-up about that.

 Even though you will receive survey links through both email and text, you should only complete one survey per day. So, do not complete the survey from your email AND the survey from your texts on the same day -- just complete one of them, because they are the same. We are sending the link through both email and text just to make sure you see it each day. 

 After your first 4 weeks in the study, you will only be asked to complete 1 survey each week for the remaining 2 months. 

 Do you have any questions about the study?

Please take a moment, if you can, to add Jena Slaski (xxxxxxxx) to your email contacts list to make sure you receive your daily survey links, so they don't get caught in your spam or junk mail folder.

 ***Click "next" below to log the patient's responses***

End of Block: Post-Survey Script

**Supplement 4: Missing Data Handling Decisions Procedures**

**Missing Data Plan:**

Analysis will be using intention-to-treat for eligible enrolled patients, maintaining the same dataset of sociodemographic and low back pain (LBP) specific registration data for each area of outcome interest (opioid prescribing and use, and pain intensity and interference (function)). Subjects who did not record any baseline or outcome data will be removed from the relevant analysis but included in LMM intention-to-treat. For example, any subjects who do not record any daily diaries cannot be analyzed in the opioid prescribing or use analysis or change in pain over time but will be included in the analysis of initial pain outcomes. Any subject who does not record an initial or follow-up Numeric Rating Scale Pain Intensity score outcomes will be excluded from acute pain analysis, but maintained in the opioid or pain interference change over time analysis.

Diary entries and item-level data will be analyzed for missing data to assess the need for imputation. When needed, imputation will be done with Last Carried Forward. Changes over time for pain and disability models will be evaluated with a Linear Mixed Model. For loss to followup and random missing data, this mixed-effects model repeated measures (MMRM) approach will be used to provide implicit imputations of missing data for continuous outcomes.

Dose, Opioid Formulation, Source Evaluation (DOSE) tool:

“New prescription” outcomes use a response to the “source” prompt of “prescribed to me for this event”. If an opioid is endorsed with the same dose and pill number on multiple days, but the source differs, any endorsement of “to me for this event” will still be considered a new prescription as a binary outcome, thus no imputation or missing data will be needed.

“Use” outcomes are any opioid endorsed with dose and brand. When multiple opioids are endorsed with source and pill number on multiple days, but not pill dose, the dose is assumed to be the most common reported by the participant if 3 or more other instances of the same are recorded.

**PAIN INTERFERENCE**

The primary pain outcome for chronic patients is clinically important change in functional pain interference using the PROMIS 8a short form over time, calculating T-scores using HealthMeasures scoring (Northwestern.edu). For low back pain, the average minimally important clinical difference using Pain Interference is 8-9 for spine patients. <https://www.ncbi.nlm.nih.gov/pmc/articles/PMC4854267/>

PROMIS scores of 10 are one standard deviation, with the numbers between 61 and 65 being most sensitive for clinical change. For Pain Interference, a score of 55 is considered mild pain, and a low back pain T-Score of 57.7 – 65.4 crosswalks to an Oswestry Disability Index (ODI) rating of “moderate”, a T-score of 65.7-71.5 to “severe”, and a score of 71.9 or more “crippled”.[2] LBP resolution for patients with chronic pain will be considered categorically as compared to TENS, defined as both a decrease of 10 or 20 AND a change in category (e.g. below mild =55 or zero disability 40.7), and a continuous variable. Exploratory outcomes of responders will be defined as reductions of 2 standard deviations (20) over the duration of followup, and a reduction in VAS to below 2.5 to compare with other rehabilitative therapies. Transition of acute to chronic pain will be evaluated based on pain of 55 or higher at 3 months (cLBP definition) for those endorsing pain <3 months at entry.

***Initial registration database review 10 days after completion of enrollment*:**

Participants were verbally screened for inclusion and exclusion criteria from an investigator guide sheet prior to informed consent, randomization and enrollment on a tablet. Supplement 3: Data Collection. The purpose of the study and opioid use reporting were discussed during informed consent.

**RE-ENROLLMENT:** For 20 subjects, the Qualtrics system did not recognize the format of the phone number or email entered at registration, resulting in failure of Qualtrics to remind patients via one or both modalities to enter that day, week, or month’s information. To fix the diary notification issue, subjects were re-enrolled remotely by the study coordinator and a new diary ID was automatically generated. During subsequent re-enrollment by the study coordinator to activate the daily reminder notifications, data fields were filled in with nonsense data or bypassed as the goal was just to activate the notification system. The intervention allocation remained that of the device dispensed at enrollment.

**Procedure:** unless noted, the ID and data from the first ID associated with the registration minimum dataset data (“Registration ID”) will be used; diary data will be merged to the registration ID from the time the re-enrollment ID is active. If duplicate diary days are entered during transition, the earlier of the two will be used. Numbers 10 9106 2783 4184 test registrations. 8560 no monthly or weekly.

Table 1. Initial registration numbers and NewID diary numbers.

| Reg ID | New ID | Enrollment | Re-E | Days to |  |
| --- | --- | --- | --- | --- | --- |
| 8962 | 9906 | 6/25/2022 | 6/27/2022 | 2 |  |
| 1525 | 5015 | 6/25/2022 | 6/28/2022 | 3 |  |
| 5393 | 2485 | 6/25/2022 | 6/27/2022 | 2 |  |
| 8491 | 2315 | 6/25/2022 | 6/27/2022 | 2 |  |
| 4050 | 596 | 6/25/2022 | 6/27/2022 | 2 |  |
| 7241 | 1435 | 10/3/2022 | 10/3/2022 | 0 |  |
| 3500 | 8134 | 10/14/2022 | 10/20/2022 | 6 |  |
| 9824 | 2826 | 10/18/2022 | 10/21/2022 | 3 |  |
| 8818 | 8653 | 12/5/2023 | 12/7/2023 | 2 |  |
| 9645 | 5370 | 1/10/2023 | 1/10/2023 | 0 |  |
| 1015 | 5033 | 1/10/2023 | 1/23/2023 | 13 |  |
| 6951 | 1725 | 2/17/2023 | 2/17/2023 | 0 |  |
| 7238 | 8422 | 2/20/2023 | 2/22/2023 | 2 |  |
| 5623 | 2396 | 3/6/2023 | 3/9/2023 | 3 |  |
| 8762 | 4268 | 4/14/2023 | 4/19/2023 | 5 |  |
| 2995 | 2805 | 5/26/2023 | 6/1/2023 | 6 |  |
| 5960 | 9088 | 8/23/2023 | 8/23/2023 | 0 |  |
| 8473 | 6618 | 10/3/2023 | 10/4/2023 | 1 |  |
| 5279 | 4807 | 10/31/2022 | 10/31/2022 | 0 |  |
|  |  |  |  |  |  |
|  |  |  |  |  |  |

**Pooling Stratified Analysis**

**ACUTE V. CHRONIC:** As prognosis, pain, and etiology differ between acute and chronic pain, the [The NIH BACPAC consortium](https://heal.nih.gov/files/2021-02/BACPAC%20Definition%20of%20cLBP%20%28BACPAC_2014%20Task%20Force%20Crosswalk%29%20%281%29.pdf) V1 Aug 4, 2020 minimum dataset includes 6 different time frames of pain, defining acute low back pain as pain lasting less than three months. Chronic pain studies have found an increased risk of persistent LBP after one year, and increased pain associated with 3 or more years of cLPB.

The FDA requested 6 months of follow-up for chronic patients, and we decided to use 3 months for acute pain participants. Participants populated identical registration data on a tablet stratifying participants into a 3 month or 6-month follow-up data collection program. After informed consent and before the participant was given the study intervention, the data collection tool prompted the enroller to verify “How long has the patient experienced ongoing back pain? (If greater than 3 months, switch to the chronic survey.)”

The enroller was then instructed to transition to the Chronic enrollment if the participant endorsed low back pain longer than 3 months. One patient was re-enrolled in the Chronic follow-up duration at this time.

| 3361 (A) | 3683 C | 12/5/2023 | 12/5/2023 | 0 |  |
| --- | --- | --- | --- | --- | --- |
|  |  |  |  |  | (acute->chronic after enrollment question. ID 3683 used throughout) |

Of the 60 patients verbally endorsing and enrolled in the Acute follow-up duration strata, on a later demographic question of “How long has low back pain been an ongoing problem for you?), 16 endorsed ongoing pain of longer than 3 months.

**Procedure:** For all outcomes in which acuity is a clinical consideration for pain, the pain duration reported in the registration minimum dataset will be used to stratify duration. As pain duration is not a recognized prognostic indicator of new opioid prescribing, the acute and chronic strata will be pooled. For consideration of completion of 3 or 6-month follow-up, adherence to the initially assigned computer data collection duration will be used, as these participants were not identified in time to extend their monthly data collection.

**Table 2: Average Baseline Pain and Disability Scores of Acute, Acute-on-Chronic, and Chronic.**

|  | NRS1 (Pain Now) | NRS2 (Past 24 hours) | Pain Interference |
| --- | --- | --- | --- |
| Acute (n=44) | 5.91 | 6.68 | 65.1 |
| Acute-on-Chronic (n=16) | 5.62 | 6.47 | 62.9 |
| Chronic (n=99) | 5.35 | 6.28 | 63.1 |

**PRIOR OPIOID USE:**

Some participants denied opioids at screening but then endorsed using an opioid in the registration minimum dataset. As many lay people do not understand which medications fall under the category of opioid, e.g. knowing that ibuprofen is not an opioid while tramadol is, a comprehensive opioid data collection tool was used in the registration dataset and the daily opioid diaries, which may account for the denial of opioids during screening but endorsing later.

**Procedure:** For all outcomes involving prior opioid use, a response of “no short-acting opioids” and “no long-acting opioids” recorded in the registration minimum dataset will be considered “opioid-naïve”. Participants endorsing one or more specific opioids and not endorsing “no short-acting opioids” and “no long-acting opioids” will be analyzed as a “prior user”. Opioid outcomes for prescribing will be followed weekly for 3 months.

**MIXED IBUPROFEN/ASPIRIN FORMULATIONS:**

In order to capture all potential types of opioids a participant might have in the house in the DOSE tool (Supplement 2), US brands, international, current and no longer prescribed were included.

To capture opioids prescribed or dispensed in other countries, a final category was made for “Other Short Acting [ex: Dihydromorphine, Nicomorphine, Oxycodone with Aspirin or Ibuprofen, Oxymorphone] “

Of the 60 enrolled in the Acute Group who verbally denied initiating opioid use for this episode of pain, 10 participants immediately endorsed those medications but no other opioids, and recorded “no short-acting opioids” and “no long-acting opioids”. On the data prompt in the enrollment tablet the words “Aspirin and Ibuprofen” were noted to start a new line:

Other Short Acting [ex: Dihydromorphine, Nicomorphine, Oxycodone with

Aspirin or Ibuprofen, Oxymorphone]

On daily opioid diaries, 30 people in the first 10 days endorsed this selection, putting in 325, 200, 400, or 600 and a number of pills correlating with 200mg/pill, dosing common for Aspirin and ibuprofen.

Oxycodone with ibuprofen or Aspirin are much less common in circulation than the frequency reported using this choice endorsement. To verify local trends, we consulted with a clinical opioid researcher at John’s Hopkins in the catchment area for the study. She denied any instance of a patient using any of the drugs in the "other short acting" option in her patient population or in the pre-op opioid weaning clinic. The study clinician contacted two subjects, who endorsed their intent to record ibuprofen.

**Procedure:** The category of “Other Short Acting [ex: Dihydromorphine, Nicomorphine, Oxycodone with Aspirin or Ibuprofen, Oxymorphone]” will be removed and the answers will not be considered in the opioid use, as the subjects were presumed to be responding to the Aspirin or Ibuprofen prompt.

***Blinded database review after completion of follow-up:***

**Exclusion/Inclusion Criteria:**

**Pain Severity:** Participants verbally endorsed the inclusion criteria of moderate to severe pain of (>=4 out of 10), the accepted level established by the International association of the Study of Pain and recently reendorsed and defended by the IMPAACT pain research guidelines.[Langford] After enrollment, participant pain recorded in the registration minimum dataset did not meet the NRS cut off inclusion criteria of the “pain now” or “pain past 24 hours” for 14 of 159 participants.

We consulted the literature to determine the proper approach when ineligible patients were mistakenly included. To ensure “**the decision to remove such patients is unbiased and not influenced by events that occurred after randomisation (and may therefore be affected by whether patients received experimental or control treatment), an independent adjudication committee blinded to treatment and outcome must systematically review each patient.**”[4]

We created an Independent Adjudication Committee by contacting colleagues on NIH PURPOSE pain listserv and prior NIDA scientific review group panels with experience in opioid research.

-A senior researcher in pediatric pain recommended including all who verbally endorsed moderate to severe pain.

-A researcher in opioid weaning noted that six participants did not reach the entry NRS of 4, but qualified as high impact chronic pain because “In the past three months, how often did you have pain?” was answered as most or every day, and Pain Interference >55s, indicating limited daily life or work activities on most days or every day.

-A Veteran’s Administration NIH pain investigator recommended excluding those who didn’t have moderate to severe pain on any metric, but also would have included four chronic pain patients who had pain for over two weeks, Pain Interference T scores >50, and endorsed the worst pain as a 3/5 or higher in the past 7 days.

-A PURPOSE pain researcher recommended completing the analysis including and excluding those who did not record NRS levels of 4 or more and reporting results of one in supplemental material.

In the most recent Back Pain Research Task Force,[5] Deyo et al recommend using the sum of the 7 day average Pain Intensity, PROMIS Physical Function and Pain Interference scores from the low back pain minimum dataset to define LBP impact ranging from 8 - 28-34=moderate, >=35 severe. They note that the cutoffs were determined in a “rather severely affected” sample of 218 patients presenting for epidural administration, and recommend “simply reporting actual scores, along with any categorization that investigators may choose.

**Procedure:** We calculated intention to treat (n=159) including all who had verbally acknowledged moderate to severe pain >=4 out of 10. For pain outcomes, we will analyze a subset of more severely affected participants with a Back Pain Research Task Force RTF >27 (n=110).

**BMI:** Exclusion criteria was set in the pilot study at “BMI>=30 or device won’t fit” based on the 45” prototype neoprene belt maximal would hold the device against the back with pressure without causing discomfort. The final 54” Neoprene belt is indicated up to BMI<50. Fit was used by clinic staff. One subject with a BMI of 60 was randomized to DuoTherm but was unable to attach the device as directed based on abdominal girth and diary use data (exclusively reported lying on the device in bed).

**Procedure:** Participant 6468 will be excluded from analysis. Additional analyses controlling for BMI will be conducted. Future studies are advised to include a calculator embedded in the programming prior to distributing a device.

**Repeat Diary Data**

For weekly or monthly surveys, if duplicate entries within hours or days are recorded the earlier of the surveys will be used if substantially equivalent (e.g. they forgot if they had filled it out and re-entered). If one survey was substantially more complete as measured by the “PERCENT” column indicating the percent of the survey which was completed, the survey with more complete data was used.

For daily surveys, if the day prior was missed and an entry is made on the following day before noon, it will be used as the previous day.

If two entries were for the same day at similar times, the earlier of the surveys will be used if substantially equivalent. If one was partially filled out, the survey with more complete data was used UNLESS the second survey added opioid information, in which case opioid data was added to prior more complete information. If the second included opioids and was just as complete, it was used.

For Example:

First diary at: 22:30 - no opioids, pain diary complete

Second entry at : 23:15 oxycodone, no pain diary

**Procedure:** Data used: Second oxycodone, First pain diary information

**Duplicate Data:** No entries were combined; 73 of 4570 were culled due to redundant triggering of the reporting system or a redundant entry.

**Duration of Opioid Use**

**Missing Data:**

**DOSE tool**: The skip-logic algorithm for the DOSE tool took participants to the next choice (dose, number, source) with a forward button or tab. When a prompt was skipped to go to the next question, missing data was assigned #NULL in the registration dataset, and -99 in diary datasets. When the back button was pressed, the endorsement of the opioid was not erased, and a #NULL was not recorded for pill dose, number of pills or source. Confirmation of an accidental endorsement could be confirmed if participants then endorsed “no opioid use”.

**Procedure:** When an opioid was endorsed a single time for a respondent without a response or missing data flag for pill dose, number, or source, AND when there were no other opioids for the participant, with 10 or more diary days recorded, the single entry was presumed to be in error and no MME were attributed.

4570 diary days recording any opioid use were recorded prior to time stamp consolidation. This happened for two entries, and the daily diaries were all filled out as “no opioid use”. (n=2, 125, 4003).

**Mg Morphine Equivalents**

The doses were calculated using the HHS morphine equivalents page. Initially fentanyl patch dosing was included at 25 ug/hr fentanyl patch X 24 hrs = 600 ug/day fentanyl = 60 mg/day oral morphine milligram equivalent. Subject matter experts on opioid use and opioid surveys noted that patches were rarely if ever prescribed for outpatient LBP exacerbations; the FDA medical device development toolkit validation review group also felt fentanyl and oral medications treating opioid use disorder were not relevant for an acute pain intervention study.

**Procedure:** MME will be calculated without Fentanyl patch use or medication assisted treatment (buprenorphine).

**Missing Data, DOSE**:

When a weekly survey denoted multiple strengths and pills taken, the average was recorded. For example, Codeine has 4.5MME for a 30mg tab. An entry from a weekly diary saying

| Codeine | 300/30,300/60 | 6 |  |
| --- | --- | --- | --- |
|  |  |  |  |

Would be averaged as 3 30mg tablets (13.5MME) and 3 60mg (27MME), for a total 50.5MME.

The response “9 or more” was calculated as 9.

When an opioid was endorsed but pill dose or pill number had #NULL or -99, the mode response for that participant was added if there were at least 3 other opioid days recorded N=4, 9120,3683,4382,7086 ).

When an opioid was endorsed with dose and pill number on multiple days, but source differed, any use of the source “to me for this event” was considered a new prescription. (n=1, 7181 had 2 entries saying “to me for this event” and 30+ endorsing “to me for another event” but was considered new prescription.

When an opioid has both a long and a short acting form, and the participant entered the same name, dose, pill number and source under both SA and LA, the assumption was that the participant only took one set and was unsure where to enter the data. MME was only recorded once. (n=2, 6933, 9120)

Reference Document 1. Inclusion/Exclusion Training Guide:


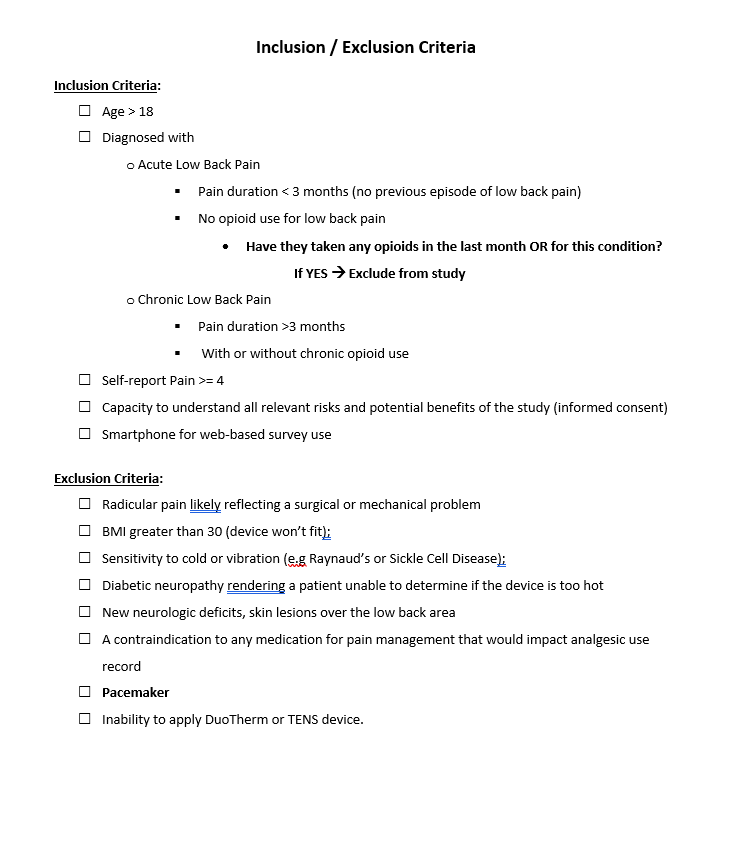


Reference Document 2:
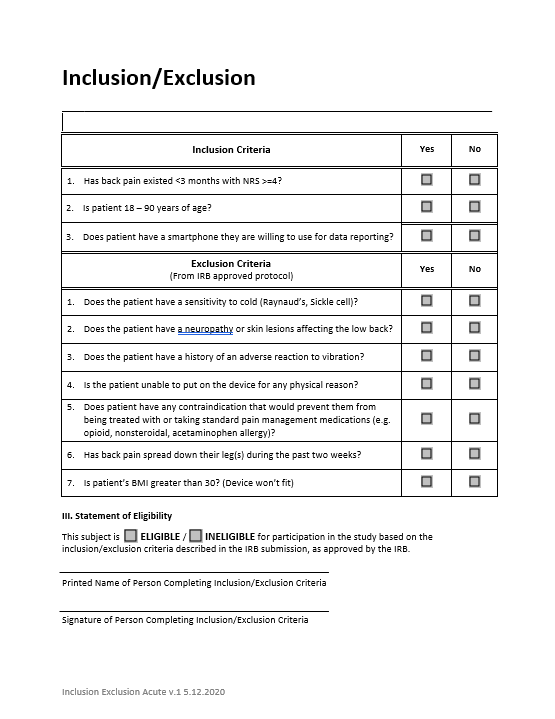


**Supplement 5: Data Sharing Statement**

**Plan to Share Individual Participant Data (IPD)?**

Yes

**IPD Plan Description**

Individual participant data that underlie the clinical results reported in any publications, after deidentification, will be provided to the HEAL NIH dataset for low back pain (BACPAC) within 12 months of publication of the results. Data will be available 36 months after publication to achieve approved aims of any researcher who provides a methodologically sound proposal. Proposals should be directed to info@mmjlabs.com. To gain access, data requestors will need to sign a data access agreement.

**IPD Sharing Access Criteria**

Automatic to HEAL Data Sharing Registry. Researchers providing approved methodologically sound proposals

**IPD Sharing Time Frame**

36 months after publication of results

**IPD Sharing Supporting Information Type**

Study Protocol – included in public eSupplement

Statistical Analysis Plan (SAP) – included in publication

Clinical databases in .csv files

**Supplement 6: Development of Predictive Neural Network for Disability Resolution**

The goal of the the study was not just to compare two different pain devices, but to understand the novel technology of the multimodal M-stim device and identify the optimal candidates for pain reduction and potential reversal of chronic pain. As an initial pivotal study of a novel device with a heterogeneous population, …

**Neural networks** are highly complex interactions of adjacent nerves and connections with predictable patterns of behavior in response to stimuli. In computer science, neural networks construct layers of “neurons” that create interactions between provided data. The parameters of the model are optimized through training of forward and backward vectors of relationships (multivariable calculus) to reach a model with the lowest error when tested against the actual data. Neural networks can predict which subject characteristics are most associated with outcomes of interest. To predict Week 13 PROMIS T-scores, a neural network was chosen because interactions between neuron features provide the possibility to discover more complex patterns within the data.

Networks vary in number of hidden layers and neurons. We had one input layer (the dataset) converted to vector form; we used 2 hidden layers with 64 neurons in the first and 32 in the second. The activation function used was rectified linear unit and the objective loss function was mean squared error (MSE). To help reduce errors, an ensemble of 5 feed-forward neural networks were created with two hidden layers. The predictions for a given PROMIS T-score were produced through the average of the models. Dropout and early stopping were used to reduce overfitting. Optimal train/test split was found to be 90/10. The model produces Week 13 PROMIS T-score predictions with a Mean Average Error of 3.82. This proves the model has significance as guessing the mean values for y_test gives a MAE of approx. 6.91. The r-squared graph is as shown:


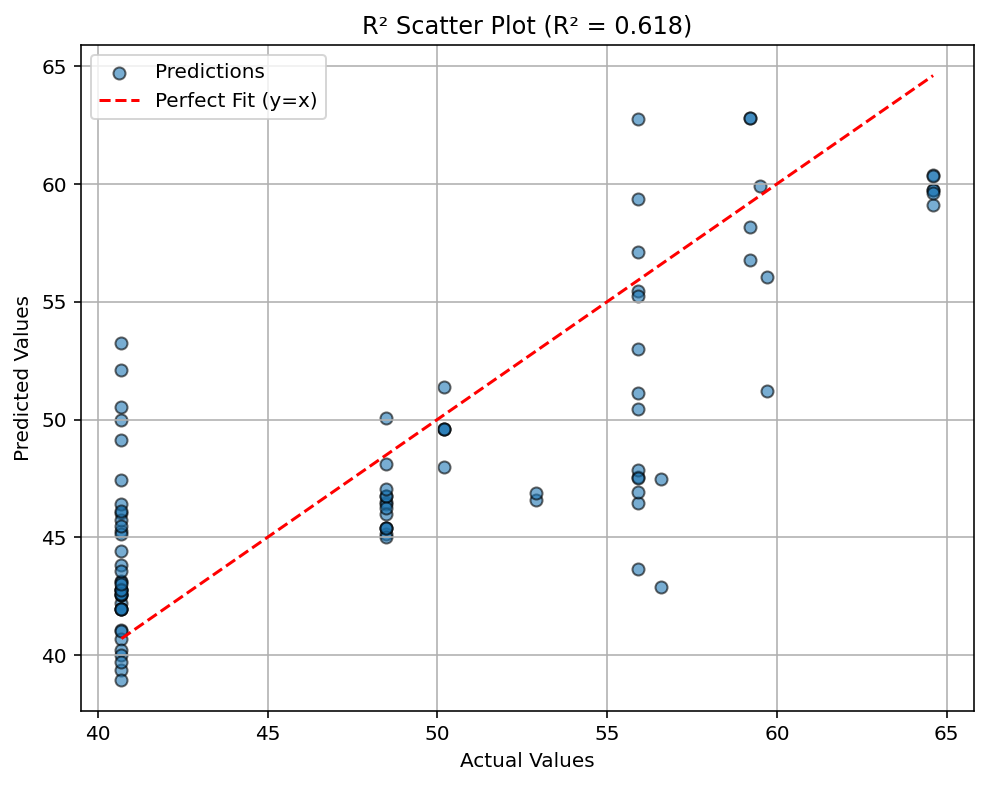


It’s clear that true values of PROMIS scores lead to a very clustered domain. The main limitation of the model is this underlying distribution of the data.

With a model that predicts PROMIS T-Scores on data of patients with significant outcomes, the feature importance can be extracted to determine what attributes most effect the model’s decision. The SHAP values are displayed below:


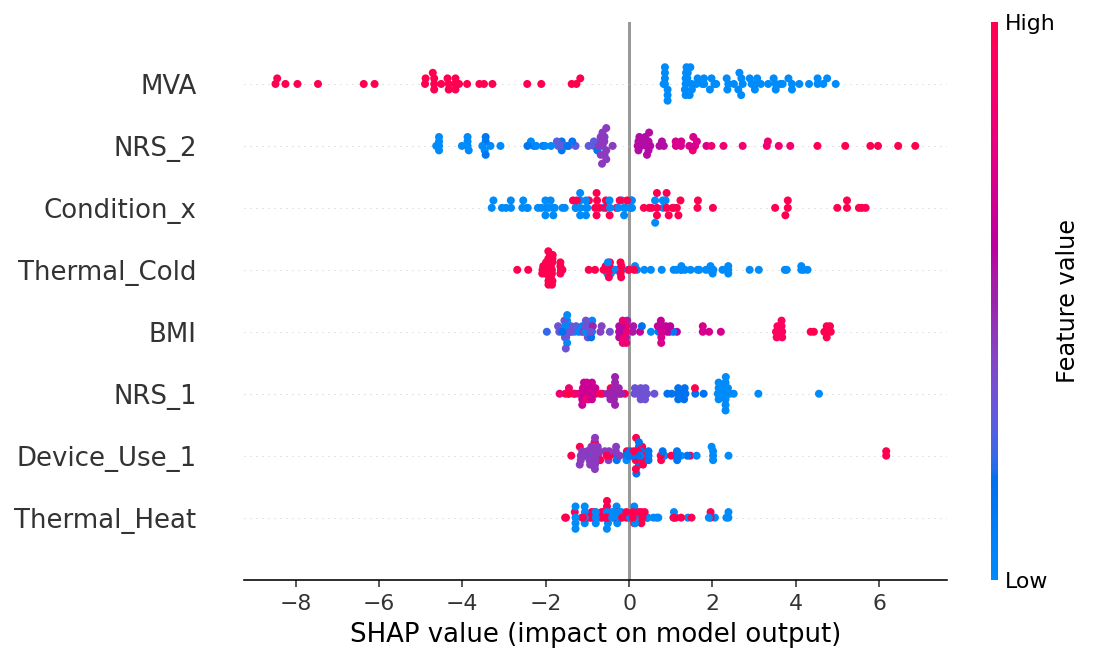


Predictions of week 13 PROMIS score for both conditions are shown, with each dot representing an individual subject.

Colors show whether the feature made a large or small impact on week 13 PROMIS scores, where negative values equate to lower ending PROMIS scores (better outcomes). For example, MVA red (1) had an MVC, and these subjects were more likely to have a better final score. blue=0 (no MVC) was highly predictive of better improvement in week 13 scores. MVA Red = Yes Blue = NO; NRS_2 = 24 hour pain intensity, continuous variable. Condition 1=blue, condition 2=red. Thermal use of cold: Red = yes used cold Blue = No use of cold; BMI continuous variable: Red high, blue low. NRS_1 Pain improvement >=3 continuous red higher. Device use

To predict what contributes to significant change in disability, only those with chronic pain (Pain_Length >= 4, meaning 6 or more months, >1 year, or >5 years) were included. the change in “pain now” was included at a higher level of pain intensity over the 13 weeks than the 10 day model. Thus, only **(ΔNRS_1 =<-3) OR NRS_2=<-2 or -3)** improvement were included in the model.

There are some immediate trends observable from how the model makes its prediction. Clearly, MVC (Motor Vehicle Collision), a binary variable, has a significant impact on predicted outcomes. Interestingly, a value of 0 (no motor accident) leads to higher predicted PROMIS T-scores. This same trend can be observed from Thermal_Cold, another binary variable. A value of 0 (no cold applied) seems to be associated with higher PROMIS T-scores. Device_Use_1 is a scalar value of time in minutes that a device was used. The model suggests that more time used decreases PROMIS T-scores. Furthermore, lower BMI seems to be associated with lower predicted PROMIS T-scores.

Some attributes seem to have mixed influence on the model, such as Thermal_heat. The clustered values support this, as well as the SHAP data that puts it at the bottom of feature importance.


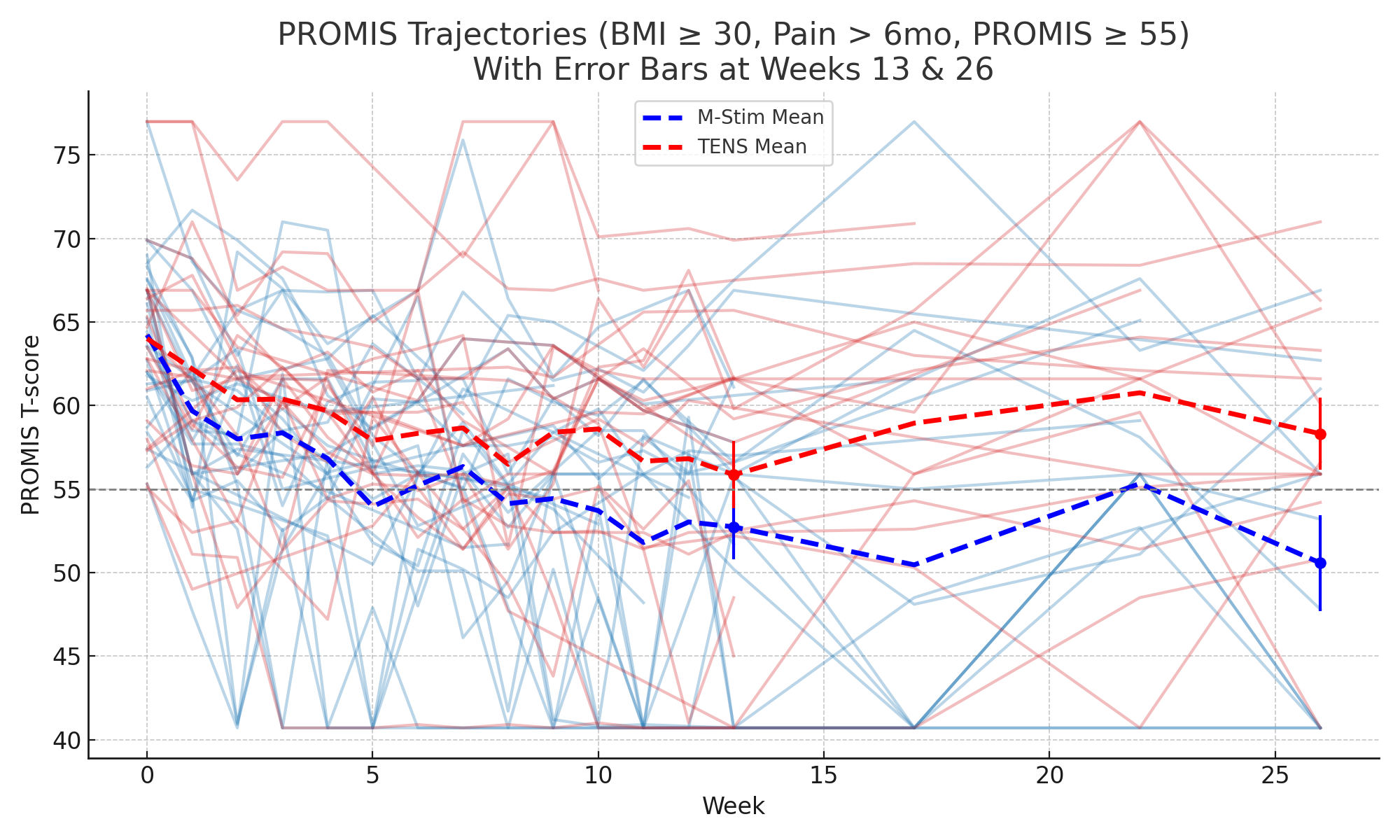
**Supplement 7: PROMIS and NRS Data**
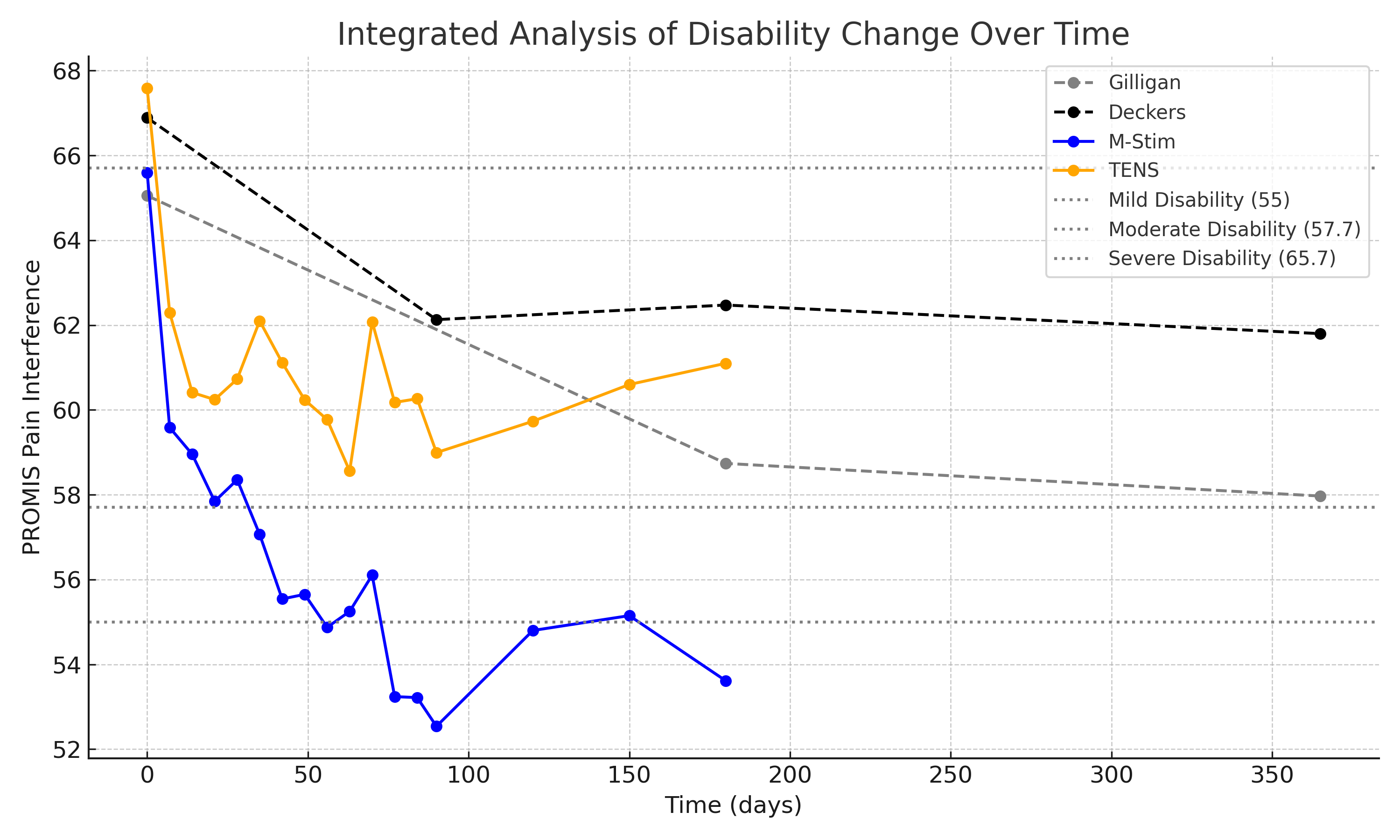


**Change in NRS**

Condition 1= M-Stim

Condition 2=TENS


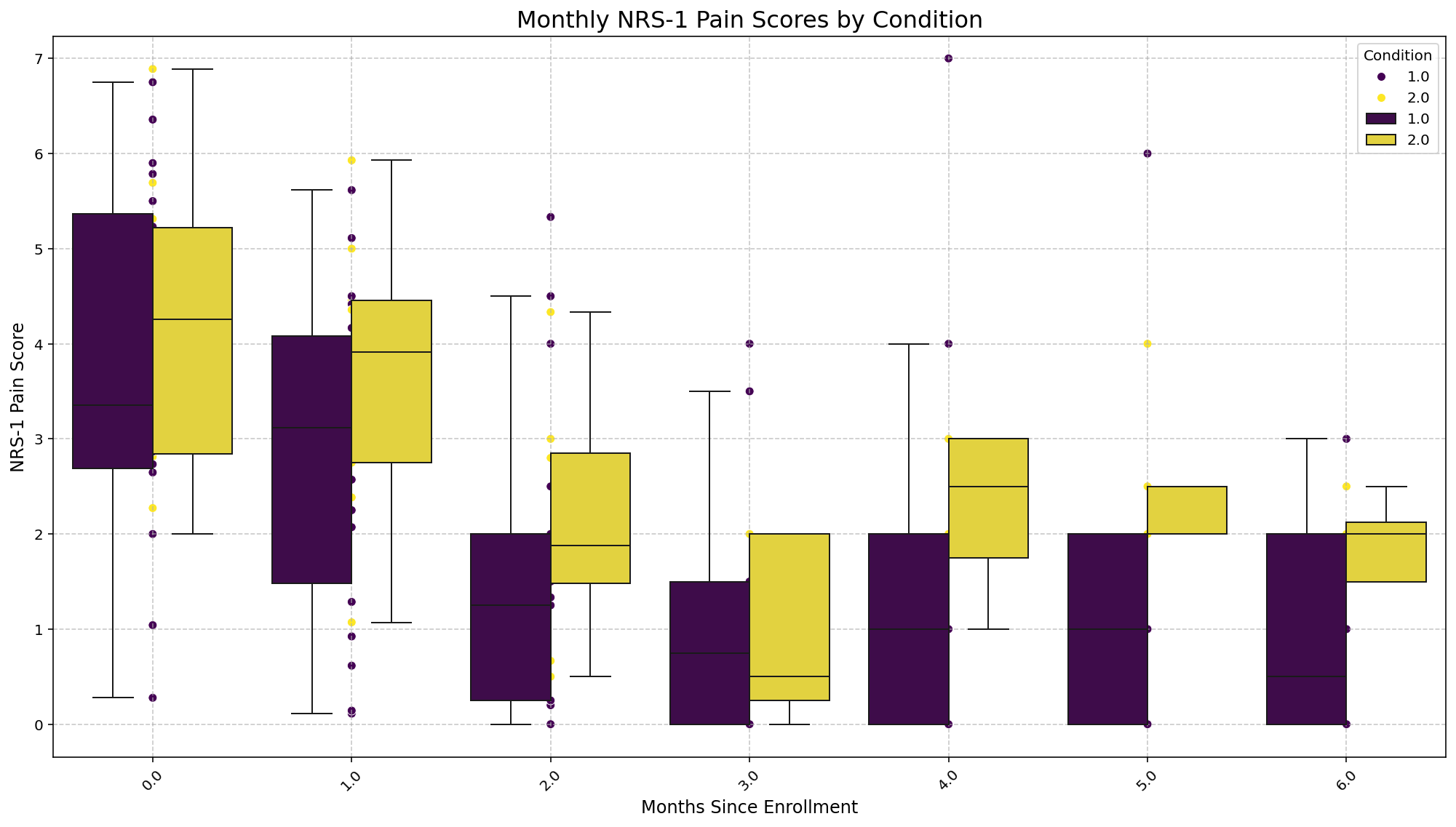


**NRS “Pain Now” Pain Intensity**

| Performance Measure | Acute M-Stim  (n=22) | Acute TENS  (n=22) | All Chronic M-Stim (n=65) | All Chronic TENS (n=50) | Chronic RTF M-Stim (n=45) | Chronic RTF TENS (n=28) |
| --- | --- | --- | --- | --- | --- | --- |
| NRS1 Baseline | 5.86 ± 2.17 | 5.95 ± 2.06 | 5.42 ± 1.97 | 5.25 ± 2.17 | 5.93± 1.86 | 6.39±1.95 |
| 30 minute NRS | 4.14[2.57]  (n=21) | 4.41[2.22] | 4.06[2.27]  (n=62) | 3.76[2.47] (n=49) | 4.59[2.33]  (n=44) | 4.96[2.33] |
| NRS Change [SD] (%) | -1.57[1.83]  (-26.8%) | -1.55[1.50]  (-26.1%) | -1.44[1.83]  (-26.6%) | -1.51[1.71]  (-28.6%) | -1.27[1.81]  (-22.6%) | -1.43[1.71] –(22.4%) |
| Day 10 NRS | 4.55[1.99] | 5.05[2.20] (n=21) | 3.88[2.5-]  (n=64) | 3.78[2.43]  (n=48) | 4.25[2.57] | 4.79[2.23] |
| Day 10 change | -1.32[1.91]  (-22.4%) p=.004 | -1.00[1.95]  (-15.1%) p=.03 | -1.55[2.36] -(-29.0%) | -1.57[2.32]  (-29.5%) | -1.59[2.36]-27.5% | -1.61[2.36]  -25% |
| Resolution (NRS ≤2.5) | 3(13.6%) | 2(9.5%) | 18(28.1%) | 18(37.5%) | 11(20.44%) | 6(21.43%) |
| Day 28 NRS | 3.14[2.34] | 4.48[2.64]  (n=21) | 3.62[1.80] | 3.44[2.47] (n=50) | 3.95[2.74] | 4.42[2.56] |
| NRS Change | -2.72[2.43] p=<.0001 | -1.57[2.87] p=0.021 | -1.80[2.45] | -1.84[2.16] | -1.89[2.55] | -1.96[2.30] |
| NRS ≤2.5 | 9(41%) | 5(23.8%) | 24(38.7%) | 18(37.5%) | 15(33%) | 5[17.9%) |
| Week 13 NRS | 2.36[2.61] | 3.38[2.50]  (n=21) | 3.02[2.15]  (n=63) | 3.04[2.64]  (n=49) | 3.16[2.80]  (n=44) | 3.86[2.89] |
| Week 13 change | -3.50[2.97] (-53.4%) | -2.67[2.50]  (-42.7%) | -2.40[2.61]  (-44.7%) | -2.22[2.95]  (-39.73%) | -2.67[2.84]  (-46.1%) | -2.54 [3.30] (-34.8%) |
| NRS ≤2.5) | 13(59%) | 8(38%) | 31(48.4%) | 26(52.0%) | 22(48.89%) | 11(39.29%) |
| Week 26 |  |  | 3.0[2.68] | 3.22[2.75] | 3.53[2.76] | 4.21[2.99] |
| Week 26 change | n/a | n/a | -2.42 | -2.04[2.89] | -2.29[2.62] | -2.18[3.15] |
| Change in NRS (%) | n/a | n/a | -39.72%  (n=63) | -33.77%  (n=49) | -38.08%  (n=44) | -33.53% |
| Resolution (NRS ≤2.5) | n/a | n/a | 28(44%) | 22(44%) | 17(37.8%) | 9(32.1%) |


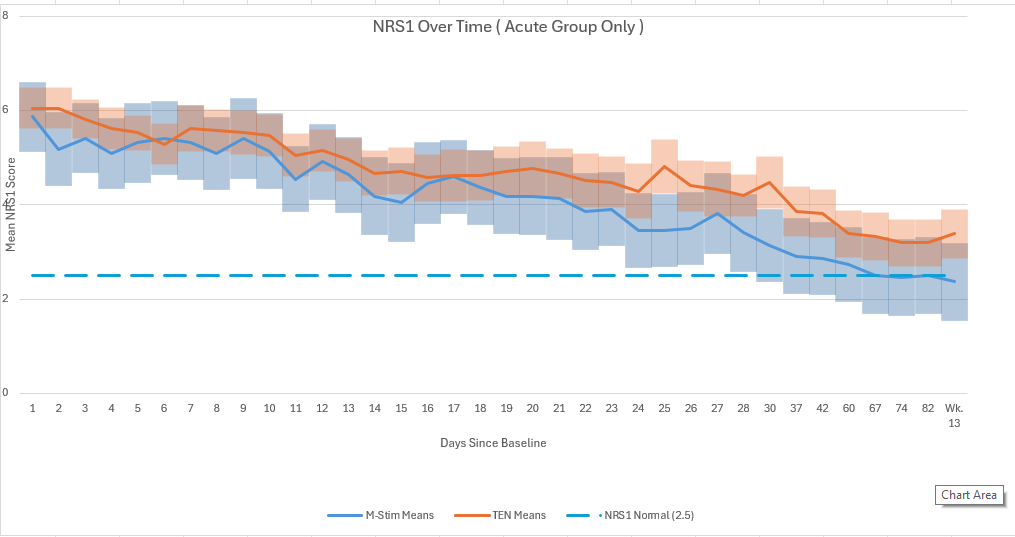


**8. References**

1. DiBenedetto DJ, Wawrzyniak KM, Schatman ME, Kulich RJ, Finkelman M. 10 kHz spinal cord stimulation: a retrospective analysis of real-world data from a community-based, interdisciplinary pain facility. Journal of pain research 2018;**11**:2929-41 doi: 10.2147/jpr.s188795 [published Online First: 2018/12/13].

2. Tang X, Schalet BD, Hung M, Brodke DS, Saltzman CL, Cella D. Linking Oswestry Disability Index to the PROMIS pain interference CAT with equipercentile methods. Spine J 2021;**21**(7):1185-92 doi: 10.1016/j.spinee.2021.02.012 [published Online First: 20210219].

3. (CMS) CfMMS. Opioid Oral Morphine Milligram Equivalent (MME) Conversion Factors table for prescription drug coverage. Secondary Opioid Oral Morphine Milligram Equivalent (MME) Conversion Factors table for prescription drug coverage 2020. <https://www.hhs.gov/guidance/document/opioid-oral-morphine-milligram-equivalent-mme-conversion-factors-0>.

4. Fergusson D, Aaron SD, Guyatt G, Hébert P. Post-randomisation exclusions: the intention to treat principle and excluding patients from analysis. BMJ (Clinical research ed.) 2002;**325**(7365):652-4 doi: 10.1136/bmj.325.7365.652.

5. Deyo RA, Dworkin SF, Amtmann D, et al. Report of the NIH Task Force on research standards for chronic low back pain. Physical therapy 2015;**95**(2):e1-e18 doi: 10.2522/ptj.2015.95.2.e1 [published Online First: 2015/02/03].
